# A Near‐Infrared Retinomorphic Device with High Dimensionality Reservoir Expression

**DOI:** 10.1002/adma.202411225

**Published:** 2024-10-10

**Authors:** Yan‐Bing Leng, Ziyu Lv, Shengming Huang, Peng Xie, Hua‐Xin Li, Shirui Zhu, Tao Sun, You Zhou, Yongbiao Zhai, Qingxiu Li, Guanglong Ding, Ye Zhou, Su‐Ting Han

**Affiliations:** ^1^ Department of Applied Biology and Chemical Technology The Hong Kong Polytechnic University Kowloon Hong Kong 999077 P. R. China; ^2^ College of Electronics and Information Engineering Shenzhen University Shenzhen 518060 P. R. China; ^3^ Institute of Microscale Optoelectronics Shenzhen University Shenzhen 518060 P. R. China; ^4^ Institute for Advanced Study Shenzhen University Shenzhen 518060 P. R. China

**Keywords:** flash memory, high dimensionality reservoir, in‐sensor reservoir computing, retinomorphic device, upconversion materials

## Abstract

Physical reservoir‐based reservoir computing (RC) systems for intelligent perception have recently gained attention because they require fewer computing resources. However, the system remains limited in infrared (IR) machine vision, including materials and physical reservoir expression power. Inspired by biological visual perception systems, the study proposes a near‐infrared (NIR) retinomorphic device that simultaneously perceives and encodes narrow IR spectral information (at ≈980 nm). The proposed device, featuring core‐shell upconversion nanoparticle/poly (3‐hexylthiophene) (P3HT) nanocomposite channels, enables the absorption and conversion of NIR into high‐energy photons to excite more photo carriers in P3HT. The photon‐electron‐coupled dynamics under the synergy of photovoltaic and photogating effects influence the nonlinearity and high dimensionality of the RC system under narrow‐band NIR irradiation. The device also exhibits multilevel data storage capability (≥8 levels), excellent stability (≥2000 s), and durability (≥100 cycles). The system accurately identifies NIR static and dynamic handwritten digit images, achieving recognition accuracies of 91.13% and 90.07%, respectively. Thus, the device tackles intricate computations like solving second‐order nonlinear dynamic equations with minimal errors (normalized mean squared error of 1.06 × 10⁻^3^ during prediction).

## Graphical abstract

Near‐infrared retinomorphic device featuring a core‐shell upconversion nanoparticle/organic material nanocomposite channel, achieving simultaneous perception and encoding information. The synergy of photovoltaic and photogating effects ultimately determines device operates as dynamic reservoir with high expression power, nonlinearity response, short‐term memory, multiple states, and large dynamic range for affordable reservoir computing‐based edge learning, therefore unleashing potential of artificial infrared perception.

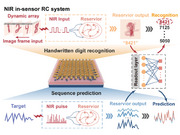

## 1 Introduction

Vision is pivotal to acquiring information, facilitating the intricate perceptual interplay between organisms and their natural surroundings, and aiding sensory engagement.^[^ ^1^, ^2^ ^]^ In human visual perception, sensory neurons in the retina initially capture continuous light stimuli. These stimuli are then transformed into discrete spike trains, which efficiently transmit the information to the visual cortex of the brain for processing.^[^ ^3^ ^]^ The discretization and inherent stochastic nature of spike‐based encoding facilitate long‐distance communication and enhance neural processing efficiency.^[^ ^4^, ^5^, ^6^ ^]^ The synergy between the retina, with seven layers of neurons, and the visual cortex, with 52 regions, is the basis of the hierarchical, compact, and adaptable learning mechanism of the brain and a fundamental artificial intelligence target.^[^ ^7^ ^]^

Biological visual perception systems have greatly inspired artificial vision research to mimic the visual pathway function for robust and general visual perception.^[^ ^8^ ^]^ In addition, infrared (IR) machine vision is crucial for various promising applications, including night vision instruments in military exercises,^[^ ^9^ ^]^ in vitro intraoperative diagnoses,^[^ ^10^ ^]^ and extending human perception beyond visible light to identify nighttime hazards.^[^ ^11^ ^]^ Conventional machine vision systems often rely on traditional deep learning frameworks, such as recurrent neural networks, for temporal processing, necessitating cumbersome training procedures like gradient descent via backpropagation through time. Such training procedures are neither scalable nor affordable on edge devices with limited battery access and form factors.^[^ ^12^, ^13^ ^]^ Beyond the traditional neural networks,^[^ ^14^ ^]^ algorithm‐wise reservoir computing (RC) is gaining attention owing to its uniqueness in minimizing the computational complexity of processing time series data. Leveraging the nonlinear reactions of a “reservoir” to input signals, RC is highly efficient.^[^ ^15^, ^16^ ^]^ “Exotic” dynamic systems are gaining momentum as reservoirs. However, the physical reservoir‐based RC system for intelligent IR perception is unsatisfactory to date owing to the following reasons.

(1) System: Physical reservoirs have low expression power compared to RC with simulated reservoirs.^[^ ^17^, ^18^, ^19^, ^20^, ^21^, ^22^, ^23^, ^24^, ^25^, ^26^, ^27^ ^]^
(2) Device: Mainstream crystalline materials, such as silicon and compound semiconductor‐based devices, do not detect IR information without a filter owing to their large band gap.^[^ ^28^, ^29^ ^]^

Additionally, intelligent devices that can simultaneously sense, filter, memorize, and process IR signals with distinct responses to light irradiations—without fast saturation and nonlinear decay in the dark—are still lacking at the device level.^[^ ^30^, ^31^, ^32^, ^33^, ^34^ ^]^ Therefore, a filter‐free IR retinomorphic device that operates as a dynamic reservoir with IR‐controlled high expression power, nonlinearity response, short‐term memory (STM), multiple states, and large dynamic range needs to be explored. This could enable affordable RC‐based edge learning and uncover the potential of artificial IR perception.^[^ ^35^ ^]^

Upconversion (UC) materials represent a class of anti‐Stocke‐shift photo‐luminescent compounds that can absorb multiple photons of longer wavelengths and emit a single photon at a shorter wavelength.^[^ ^36^ ^]^ The core‐shell upconversion nanoparticles (UCNPs) based on lanthanide ion (Yb^3+^, Er^3+^)‐doped NaYF_4_ have a narrow absorption band centered at 980 nm owing to the 4f‐4f orbital electronic transition with concomitant wave functions in the lanthanide ion, transforming two or more near‐infrared (NIR) photons into visible photons, thereby minimizing non‐absorption energy losses in photonic devices. Subsequently, UCNPs have been widely used in advanced optoelectronics and multimodal imaging within the NIR region. They are essential components in diverse photonic applications scenarios, spanning in vivo treatments, biomedical imaging tools, and optoelectronic and optogenetic devices, owing to their precise emission bandwidth, exceptional photochemical durability, and substantial anti‐Stokes shift capabilities, which can extend up to several hundred nanometers.^[^ ^37^ ^]^ However, the limitations—subdued photo‐absorption capacity and insulating UCNPs—hinder their photon‐electron conversion efficiency. Self‐assembled core‐shell UCNPs with a hierarchical design offer diverse nanophotonic solutions that retain all the qualities of conventional UCNPs while introducing specific features.^[^ ^38^, ^39^ ^]^ The electronic and physicochemical structures of UC systems can be aligned for nanophotonic control of UC or to generate new functionalities. For instance, UCNPs are vital in drug/gene delivery and photonic diagnostics by coating non‐epitaxial silica or polymer shells that can capture, encapsulate, or compound drugs.^[^ ^40^, ^41^ ^]^

Herein, we report a NIR light‐modulated retinomorphic device that simultaneously perceives and encodes narrow IR spectral information (at ≈980 nm) based on a three‐terminal transistor with core‐shell UCNPs@SiO_2_/poly (3‐hexylthiophene) (P3HT) nanocomposites channel. The P3HT semiconducting layer reabsorbs the high‐energy photons emitted from UCNPs@SiO_2_ by absorbing the NIR, generating more excitons. In addition, the SiO_2_ shell of the UCNPs@SiO_2_ nanostructure used was self‐assembled onto the UCNP core during the synthesis to directly function as a tunneling dielectric layer. It is a suitable well for photoinduced charge trapping. Based on the history of optical inputs, synaptic reactions were observed, exhibiting asymmetric relaxation, achieving an excellent expression power for the UCNPs@SiO_2_/P3HT transistor reservoir. Furthermore, the synaptic response heavily depends on the electrical programming time, which is a feature of photon‐electron‐coupled dynamics. This was used to achieve high dimensionality in a single UCNPs@SiO_2_/P3HT transistor. The synergy of the photovoltaic and photogating effects influences the nonlinearity and high dimensionality of the RC system under narrow‐band NIR irradiation. Multilevel data storage (≥8 levels), high stability (≥2000 s), and durability (≥100 cycles) can be achieved in the UCNPs@SiO_2_/P3HT transistor by manipulating the programming time of the device in the dark. Our NIR in‐sensor RC system accurately identified static and dynamic handwritten digit NIR images, achieving recognition accuracies of 91.13% and 90.07%, respectively. Furthermore, abundant electrically/optically tunable reservoirs within the UCNPs@SiO_2_‐based device enable the system to handle more complex tasks. For instance, the errors in RC tasks for predicting a second‐order nonlinear dynamic equation were minimal, with a normalized mean squared error (NMSE) of 1.06 × 10⁻^3^. This underscores the potential of our NIR in‐sensor RC system for predicting and computing sequential tasks.

## 2 Results and Discussion

### 2.1 Intelligent IR Perception System

**Figure 1:**
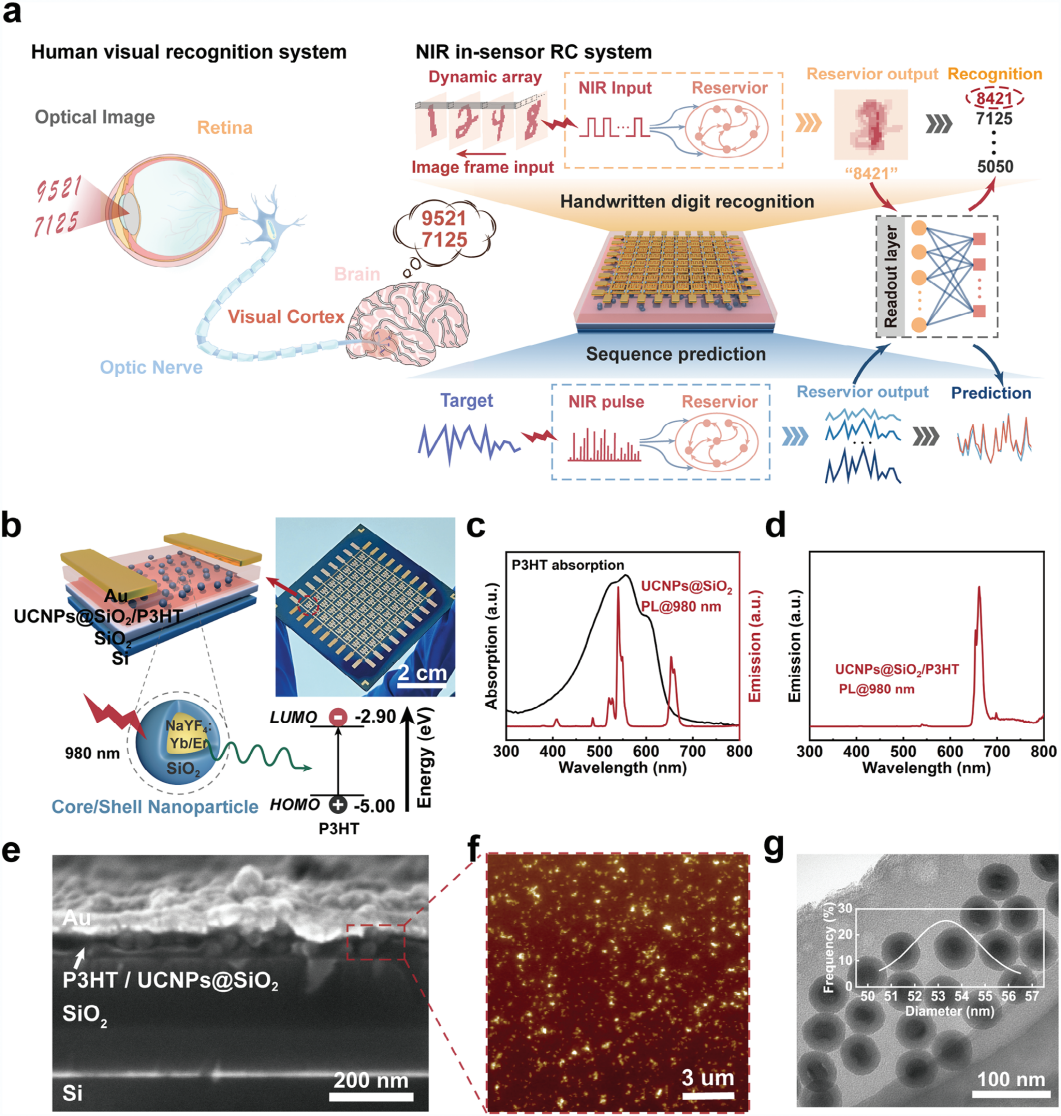
Implementing the NIR in‐sensor RC system via a single device. a) Schematic representation of the human visual recognition system (left panel) and the NIR in‐sensor RC system (right panel). b) Schematic representation of the structure of the device and RET process from UCNPs@SiO_2_ to P3HT. c) Absorption spectrum of the P3HT film and the PL spectrum of UCNPs@SiO_2_. d) The PL spectrum of UCNPs@SiO_2_ within the P3HT film, verifying the existence of RET. e) Scanning electron microscopy image of a cross‐sectional area in the UCNPs@SiO_2_‐based device. f) Atomic force microscopy image of the films in the UCNPs@SiO_2_‐based device, presenting the uniformity of the overall UCNPs@SiO_2_ distribution. g) Transmission electron microscope image of UCNPs, substantiating the core/shell structure of nanoparticles; inset is the size distribution of UCNPs@SiO_2_.

The biological visual pathway, comprising the retina, optic nerve, and visual cortex (**Figure** **1**a), underpins the physiological mechanisms of vision. Photoreceptors in the retina transduce color, shape, and brightness information into electrical signals. These signals are encoded as action potentials (spikes) by the ganglion cells and relayed to the visual cortex via the optic nerve. Distinct processing streams and the ventral and dorsal pathways in the visual cortex are specialized for motion perception and context understanding, respectively.^[^ ^42^, ^43^ ^]^ These pathways integrate information from the lower visual areas, recognizing and classifying complex visual stimuli. We designed an intelligent IR perception system that mimics the functionality of the biological visual system (Figure 1a,b). The system uses UCNPs@SiO_2_ as photoreceptors, converting NIR at 980 nm into detectable yellow‐green light at 520 and 545 nm in the semiconductor layer. The programmable nonlinear dynamics of the P3HT organic layer allow for processing received information and mapping it into a high‐dimensional nonlinear space. Finally, RC with low training requirements facilitates recognizing and classifying the encoded information. Therefore, this design embodies the perception‐transmission‐processing capabilities of the human visual pathway in a single device. Furthermore, its functionality is extended to the NIR spectrum, enabling the detection and processing of information that is invisible to the naked eye. We demonstrate the abilities of the system using handwritten digit recognition and sequence prediction tasks.

Our intelligent IR device adopts a bottom‐gate top‐contact architecture (Figure 1b). A silicon substrate with a 300‐nm oxide layer functioned as the gate terminal and dielectric layer. The active layer was a composite of P3HT doped with equal concentrations of NaYF_4_:Yb, Er, and UCNPs@SiO_2_. The directly solution‐processed active layer blurs the boundary of the semiconductor layer, floating gate, and tunneling dielectric layer, reducing the processing time and number of fabrication steps. P3HT functions as a semiconducting and visible‐light‐sensitive layer. The core of the UCNPs functioned as an IR‐light‐sensitive layer and a charge‐trapping site, whereas the silica shell acted as a tunneling dielectric layer.

Upon NIR excitation at 980 nm, UCNPs@SiO_2_ emitted light at ≈520, 545, and 650 nm (Figure 1c). The p‐Type P3HT was used as the organic semiconductor layer because of its low fabrication cost, high flexibility, and ease of solution processing.^[^ ^44^, ^45^ ^]^ The strong absorption band of P3HT (400–700 nm) overlapped with the dominant UCNPs@SiO_2_ emission peak (≈545 nm), promoting efficient radiative energy transfer (RET) (Figure 1c). This was further confirmed by quenching the 520 and 545 nm peaks and the selective amplification of the 650 nm emission in the presence of P3HT (Figure 1b,d). A cross‐sectional field‐emission scanning electron microscopy (SEM) image reveals a uniform particle size dispersed in the P3HT layer (Figure 1e). Atomic force microscopy (AFM) images confirmed the smooth surface of the P3HT film with uniform UCNPs@SiO_2_ distribution (Figure 1f; Figure S1, Supporting Information). The average roughness of the P3HT/UCNPs@SiO_2_ film was ≈8.26 nm. The transmission electron microscopy (TEM) image revealed the core‐shell structure of UCNPs@SiO_2_ with a homogeneous size distribution. Statistical analysis (inset of Figure 1g; Figure S2, Supporting Information) revealed that the average diameter of UCNPs@SiO_2_ was ≈53.45 nm, and the average shell thickness of SiO_2_ was ≈12.48 nm. This self‐assembled tunneling dielectric layer is moderately thick to ensure fast device operation and reliability. The distribution data were well‐fitted using a Gaussian function. Additionally, the P3HT/UCNPs@SiO_2_ device possesses flexible and wearable characteristics, further expanding its application (Figure S3, Supporting Information).

### 2.2 Electrical Programming of the IR Intelligent Device

**Figure 2:**
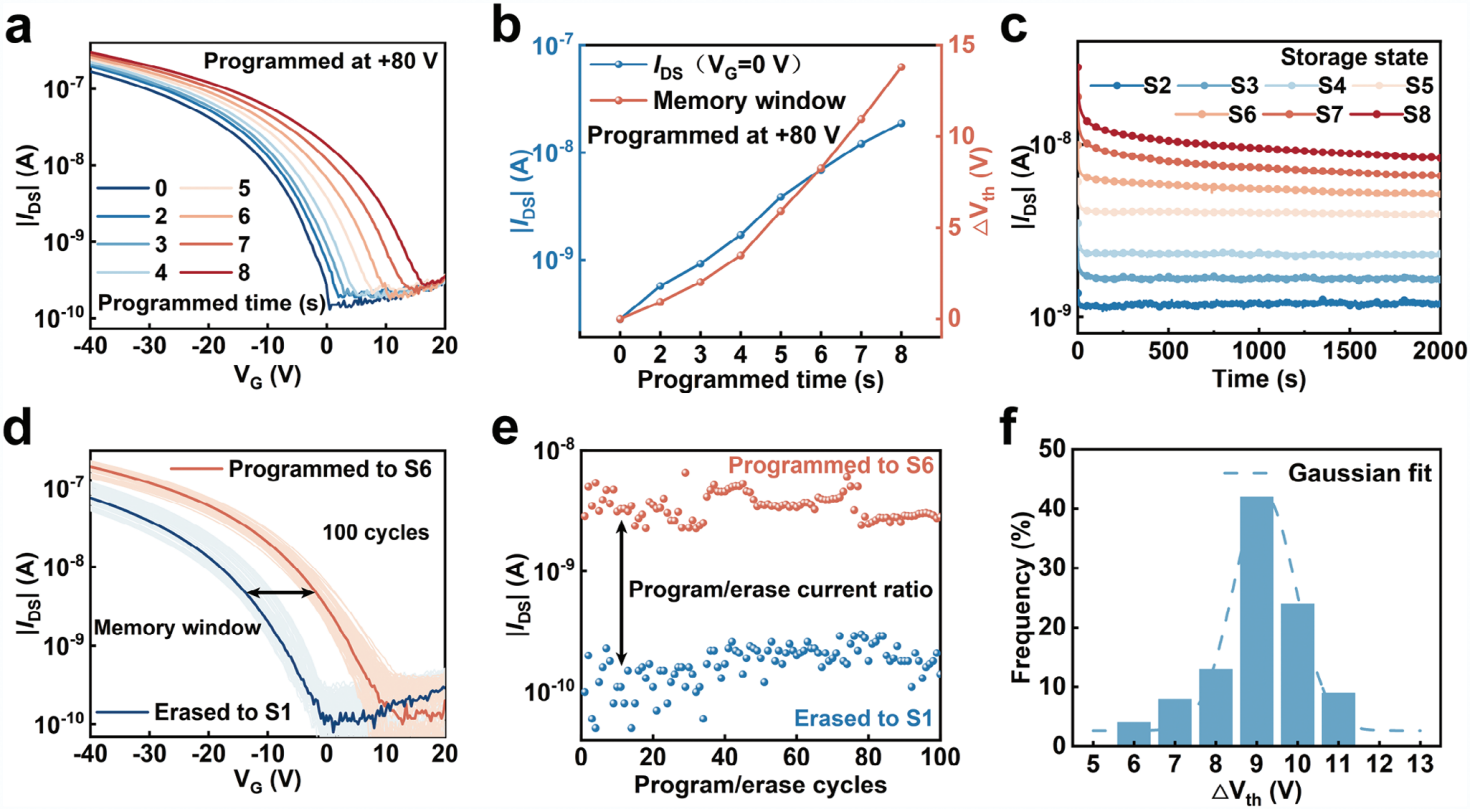
Electrical multilevel data storage characteristic of the UCNPs@SiO_2_‐based device. a) Transfer curves of the device after programming and erased operation (*V*_DS_ = –20 V). The transfer characteristic curve of the device shifted in the positive direction with the progressing programmed time because of the enhanced carrier capture state. b) Drain current and memory window of the device after different programmed times. Prolonged programmed time leads to a wider memory window. c) Retention characteristics of the multilevel device (*V*_G_ = −2 V, *V*_DS_ = −20 V). d) Transfer curves for 100 cycles of the program/erase operations. e) Endurance of the current state of the device with repetitive program/erase operations (*V*_G_ = 0 V, *V*_DS_ = −20 V). f) Gaussian statistics of memory windows for 100 cycles of programmed/erased operations.

The programming time considerably affects the storage capacity of flash memory devices. Here, we investigate its impact by applying a positive gate voltage of +80 V for various durations and measured the transfer curve at a drain bias of –20 V. A substantial rightward shift was observed in the transfer curve with increasing programming time (0–8 s) (**Figure** **2**a). After applying a positive bias to the gate electrode, the transfer curve shifted in the positive voltage direction, confirming that UCNPs@SiO_2_ trapped electrons. The trapped charge carriers transferred from P3HT screen the gate and regulate the conduction in the channel. Consequently, the threshold voltage (*V*_th_) increases from –0.28 V (initial state) to 0.64, 1.73, 3.20, 5.62, 7.99, 10.67, and 13.54 V—a distinct multilevel response. The current level of the P3HT/UCNPs@SiO_2_ device at a fixed voltage was hierarchical, highlighting its potential for multilevel data storage. Figure 2b reveals a progressive increase in the drain current (|*I*_DS_|) at a gate voltage (*V*_G_) of 0 V and memory window (Δ*V*_th_, which is defined as *V*_th, programmed state_ –*V*_th, initial state_), with extended programming time. After programming for 8 s, |*I*_DS_| exhibited a 67‐fold enhancement compared to its initial state with a 13.8 V memory window. Here, the initial state was S1, and the subsequent states achieved with programming times of 2, 3, 4, 5, 6, 7, and 8 s were denoted as S2, S3, S4, S5, S6, S7, and S8, respectively. Figure 2c illustrates the retention characteristics of the programmed states. Following the programming to S2–S8, the current level at a fixed voltage initially deteriorated but subsequently stabilized, exhibiting reliable retention behavior across multiple states for over 2000 s, owing to the reasonable thickness of the silica shell. Afterward, the endurance of the flash memory was assessed via 100 repeated write/erase cycles (write/erase voltages: +80/−80 V, pulse width: 6 s). The transfer curves acquired after each cycle (Figure 2d) confirm the preservation of reliable programmed and erased states. The |*I*_DS_| ratio remained consistently above one order of magnitude, indicating a stable cycle operation (Figure 2e). In evaluating memory window fluctuations during the endurance test, a statistical analysis (Figure 2f) revealed a Gaussian distribution, with 87% of the cycles having a memory window within the expected range (7–10 V).

### 2.3 The Wide Variation in Synaptic Response under NIR Irradiance

**Figure 3:**
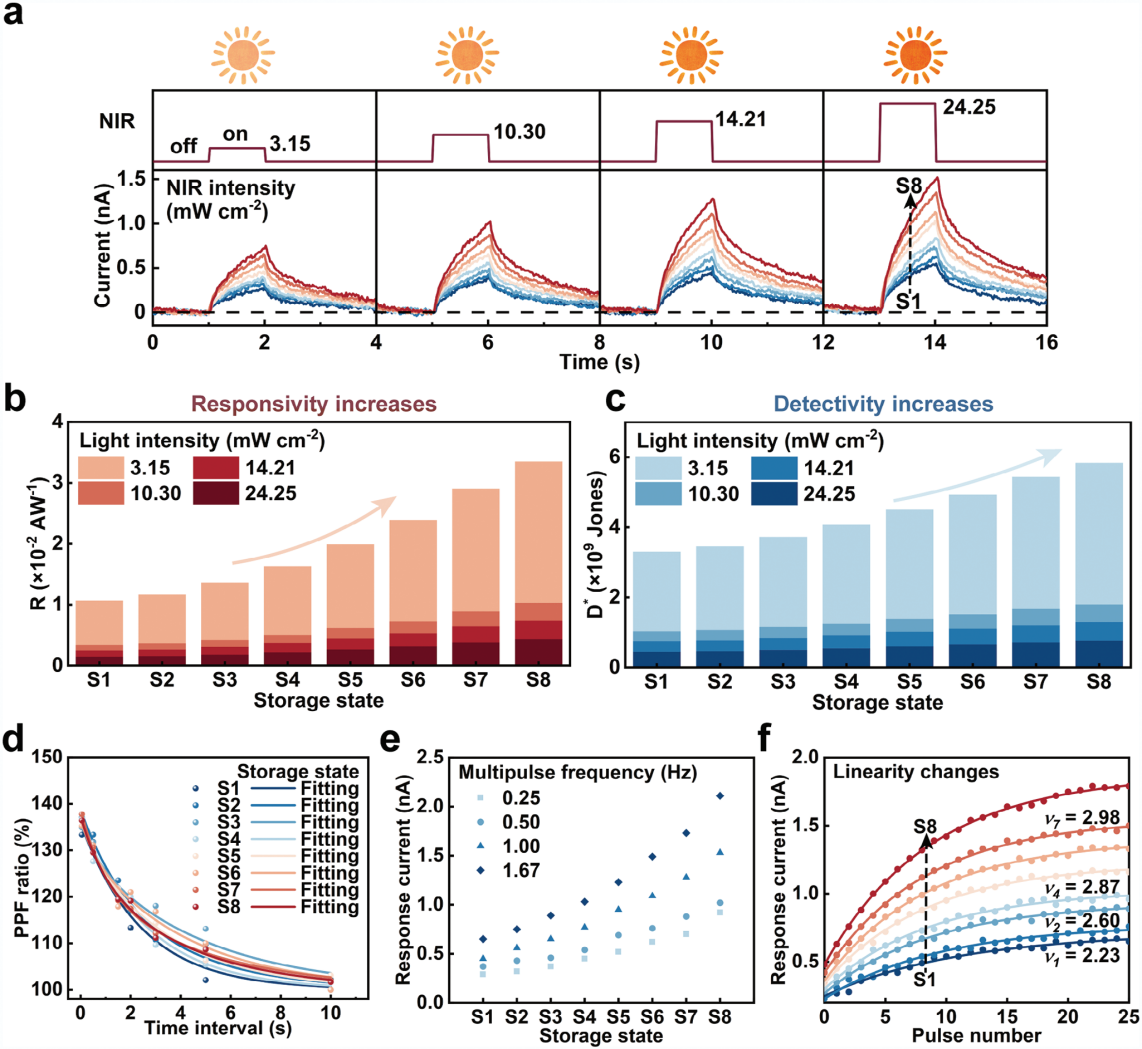
Rich optical response characteristics of the UCNPs@SiO_2_‐based device under NIR irradiation. a) The current response after applying NIR pulse of varying intensities (3.15, 10.30, 14.21, and 24.25 mW cm^−2^; 1 s pulses) under various device storage states. b,c) R and $D^{*}$ characteristics of the distinct device storage state under various light intensities. d) PPF ratio as a function of the pulse interval, with a fixed pulse width of 1.0 s and a light intensity of 24.25 mW cm^−2^ and the storage state of S1 to S8. Each storage state enables the achievement of a maximum PPF ratio of ≈140%. e) Applying 10 pulses (0.5 s, 24.25 mW cm^−2^, with frequencies of 0.25, 0.5, 1, and 1.67 Hz) under various storage states reveals that prolonged programmed time and high pulse frequency lead to a substantial current accumulation effect. f) Response current *I* as a function of light pulses number applied to device under different storage states. To evaluate the linearity in the light‐mediated potentiation behavior, fitting curves according to Equation (4) are introduced.

A broad range of synaptic responses are used to attain a heightened capacity for expression, facilitating effective information processing. The P3HT/UCNPs@SiO_2_ device with different programmed states has rich photoinduced characteristics under 980 nm laser illumination at a fixed *V*_DS_ of −10 V and *V*_G_ of −10 V. Here, we investigate the synergy of NIR irradiation and electrical programming operations (S1−S8) on the synaptic response. We used a combined signal generator and a 980 nm laser setup for precise characterization (Figure S4, Supporting Information). **Figure** **3**a reveals that a typical short‐term potentiation can be obtained when the device at S1 is exposed to an NIR pulse with 3.15 mW cm⁻^2^ intensity for 1 s. In its initial state, the device exhibited a peak current of 0.29 nA. Increasing light intensity from 3.15, 10.30, and 14.21 mW cm^−2^ to 24.25 mW cm^−2^ progressively enhances the peak current response from 0.29 to 0.5 nA owing to the increased photogenerated carriers. Furthermore, a more pronounced current response was observed by adjusting the storage state from S2 to S8 via an electrically programmed operation (increasing the programming time), even under the same NIR irradiance. The maximum current increases from 0.29 nA (initial state—S1) to 0.75 nA (S8) with a light intensity of 3.15 mW cm⁻^2^. Further analysis (Figure S5a,b, Supporting Information) revealed that the irradiance and programming time modulated the peak current and relaxation time. The device had the peak current and the longest relaxation time with a programming operation of 8 s and an IR irradiance of 24.25 mW cm⁻^2^.

We introduced two key parameters to evaluate the NIR response of the device: the photoresponsivity (R), quantifying the light response efficiency, and the photodetectivity ($D^{*}$), reflecting the device's ability to detect weak light signals.^[^ ^46^ ^]^ They are defined as follows:

(1) $$R=\frac{I_{ph}}{P_{inc}S}$$

(2) $$D^{*}=\frac{\sqrt{A}R}{\sqrt{2qI_{d}}}$$

where $I_{\mathrm{ph}}$ is the photocurrent; $P_{\mathrm{inc}}$ represents the intensity of incident light; S is the channel area; A indicates the effective device area; q is the elementary charge; and $I_{d}$ is the dark current. Figure 3b,c reveal the dependence of R and $D^{*}$ on the irradiation and programming time. Adjusting the programmed state significantly enhanced R, particularly under weak NIR illumination. When the device is at S8 with a low irradiance—3.15 mW cm⁻^2^, R reaches a maximum of 3.35 × 10⁻^2^ A W^−1^, which is a 3.14‐fold increase compared to that at S1. $D^{*}$ followed a similar trend. At S8, the device had a 1.77‐fold improvement in weak light detectability compared with that at S1. Figure S6a (Supporting Information) depicts the current response to NIR pulses of varying durations (at a constant intensity of 24.25 mW cm⁻^2^). As expected, the response current increased with prolonged illumination and programming times (Figure S6b, Supporting Information). Relaxation time analysis revealed that the NIR illumination time impacts the current decay more than the electrical programming operation (Figure S6c, Supporting Information). R changed negligibly under NIR irradiance with different pulse widths for devices in the same programmed state. Conversely, increasing the electrical programming time affected R substantially (Figure S6d, Supporting Information). This suggests that the programming time primarily influenced the light sensitivity and not the irradiation pulse width. Furthermore, the device displayed stronger light‐detectability for shorter optical pulses (Figure S6e, Supporting Information).

Adjusting the interval time (Δt) between dual pulses induces the paired‐pulse facilitation (PPF) of the device (Figure S7, Supporting Information). The PPF ratio, calculated as *A*_2_ */A*_1_, increases with decreasing pulse interval. The relationship between PPF ratio and pulse interval can be effectively modeled using a double exponential decay function.

(3) $$PPF\;ratio=1+C_{1}e^{-\frac{\Delta t}{\tau_{1}}}+C_{2}e^{-\frac{\Delta t}{\tau_{2}}}$$

where $C_{1}$ and $C_{2}$ are the facilitation ranges and $\tau_{1}$ and $\tau_{2}$ are the fitting values. All eight storage states of the device modulated the PPF ratio from ≈100% to 140% (Figure 3d). Figure 3e reveals the short‐term potentiation response at different programmed states under stimulation with ten consecutive NIR pulses of varying frequencies (0.25–1.67 Hz). A more pronounced cumulative effect was observed across the various storage states as the pulse frequency increased, leading to a progressively higher response current. This suggests the potential for enhanced memory capabilities with increasing stimulus frequency. Further investigation of the optical response nonlinearity of the device is presented, and its relationship with the storage state under 25 successive light pulses is analyzed. Subsequently, we modeled the evolution of the response current with the number of pulses using the following formula.^[^ ^47^ ^]^ This analysis provides valuable insight into the temporal dynamics and memory‐dependent behavior of devices under repeated optical stimulation.

(4) $$I=I_{0}\;\left(1-e^{-vp}\right)+I_{min}$$

where p is the normalized completed pulse parameter (25 pulses), v is the shape factor that depends on the linearity of the response potentiation characteristic, $I_{\min}$ is the initial current, and $I_{0}$ is a fitting parameter representing the dynamic response range I. Our analysis revealed a relatively low v value, indicating response curve possesses good linearity. The device had an expanded response current range at the expense of linearity after prolonged programming, with a fixed NIR pulse of 0.2 s width, 0.2 s interval, and light intensity of 24.25 mW cm⁻^2^ (Figure 3f; Figure S8, Supporting Information). Physical reservoirs exhibit various nonlinear mapping behaviors, which are reflected in the differences between the nonlinear values fitted by programming the device for different storage states. The results further corroborate that physical reservoirs possess a high‐dimensional reservoir expression through electrical programming operations. These findings further demonstrate the capability of the devices to generate wide variations and high expression ability ensure efficient information processing in RC frameworks.

### 2.4 Operating Mechanism of the UCNPs@SiO_2_‐Based Device

**Figure 4:**
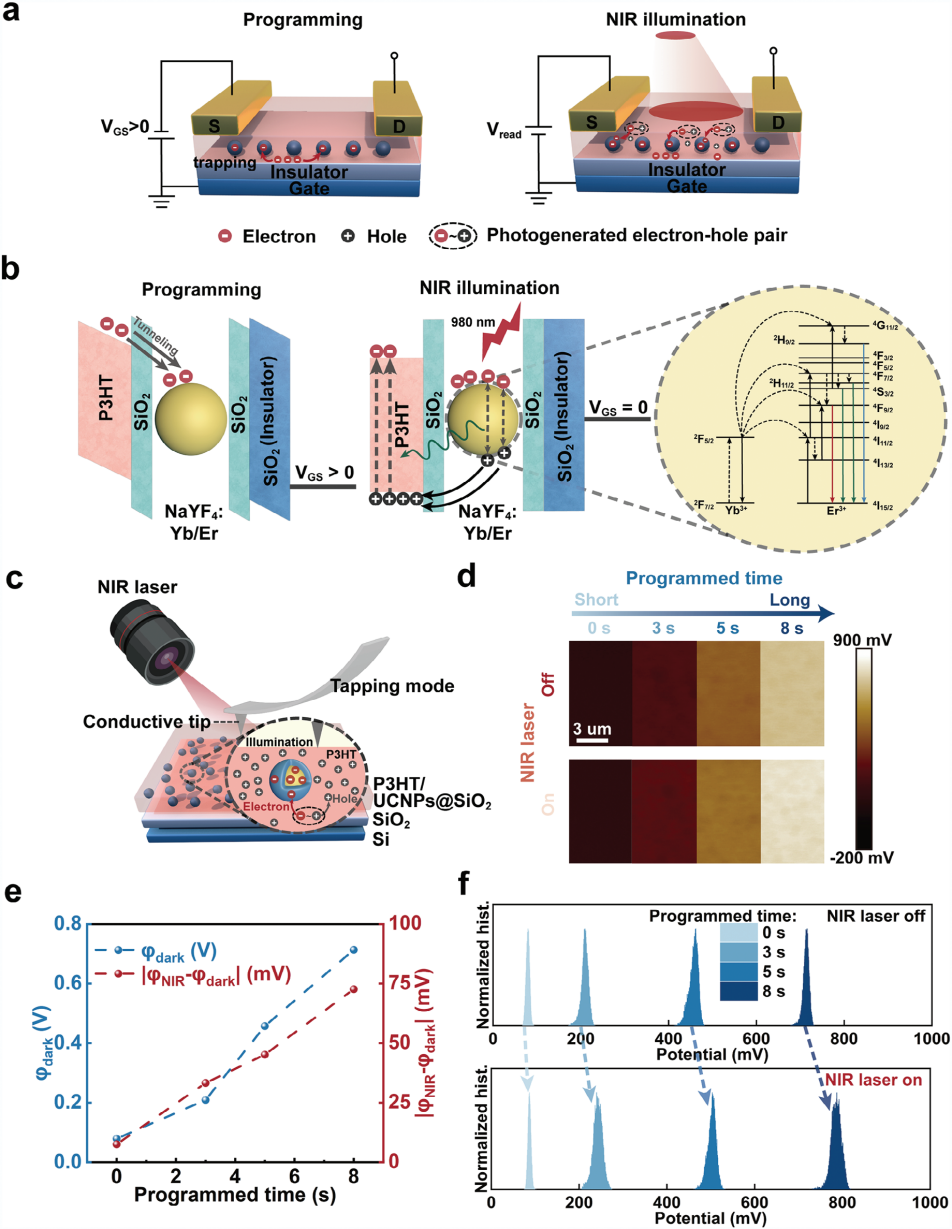
The operational mechanism of the UCNPs@SiO_2_‐based device. a) Schematic diagrams of the device during electrical programming and NIR illumination. Negative voltages are applied to gate and drain terminal to determine the current state of the device under NIR illumination. b) The energy scheme of the device during an electrical programming behavior and NIR illumination operation. The yellow area indicates the energy level of UCNPs and the working mechanism under NIR illumination. c) Scheme of the KPFM for the electrical nanotechnology of the P3HT/ UCNPs@SiO_2_ film. The scanning area is 5 µm × 10 µm. d) The surface potential of the P3HT/ UCNPs@SiO_2_ layer before and after applying various programmed times and NIR illumination recorded using KPFM. e) The trend of surface potential in the dark environment (*φ*_dark_) and the difference before and after NIR illumination (|*φ*_NIR_‐*φ*_dark_|) with increasing programmed time. f) Surface potential distribution derived from (d).

**Figure** **4**a illustrates the operating mechanism of the UCNPs@SiO_2_‐based device electrically operated in the dark. We created a charge storage interface between SiO_2_ and UCNP. During programming, the gate terminal can be used to set the memory state. A sufficiently large positive bias (*V*_G_ ≥ +80 V) drives electrons from the P3HT layer to the tunnel through SiO_2_, which is eventually trapped by the UCNPs core or interfaces. These captured electrons generate a built‐in internal electric field that accelerates hole accumulation in the semiconductor layer, consequently inducing a positive threshold voltage shift.

The tunneling dielectric layer is pivotal for charge‐trapping memory devices, crucially influencing performance metrics and reliability standards. In our study, a silica shell was introduced as the tunneling dielectric layer. The optimization of shell thickness was achieved through a delicate balance struck between enhancing programming speed and ensuring optimal data retention. According to the energy‐level diagram of the UCNPs@SiO_2_/P3HT device, the silica shell blocked the trapped electrons, functioning as a potential well and ensuring long‐term memory storage. The trapping and de‐trapping processes are reversible and repeatable. A sufficiently large negative *V*_G_ released the trapped electrons from the UCNPs@SiO_2_ back to the semiconductor layer, erasing the stored data and resetting the device. Programming voltages induced a negligible shift in the threshold voltage (Figure S9, Supporting Information), confirming the dominant role of UCNPs@SiO_2_ electron capture in programming operations.

The rich photoinduced properties of the device stem from the synergistic interaction between the RET of the UCNPs and electrical control of its internal state. Figure 4b depicts the RET using a UCNP energy‐level diagram.^[^ ^48^ ^]^ NIR photons are absorbed by the Yb^3+^ ions, which transfer energy to nearby Er^3+^ ions. Subsequently, the excited Er^3+^ ions rapidly decay via non‐radiative transitions to lower energy levels (^2^H_11/2_ and ^4^S_3/2_) and emit green light through radiative transitions back to the ground state (Figure 1b). The green emission is ascribed to the ^2^H_11/2_ → ^4^I_15/2_ and ^4^S_3/2_ → ^4^I_15/2_ transitions of Er^3+^ ions; conversely, the blue and red emissions are attributed to the ^2^H_9/2_ → ^4^I_15/2_ and ^4^F_9/2_ → ^4^I_15/2_ transitions, respectively.^[^ ^49^ ^]^ The UCNPs@SiO_2_ emission spectrum perfectly aligned with the absorption range of P3HT (Figure 1c,d), facilitating efficient RET. This is further supported by the shorter photoluminescence (PL) decay observed in P3HT/ UCNPs@SiO_2_ composites compared to that of UCNPs@SiO_2_ alone (Figure S10, Supporting Information). The shorter PL lifetime of the composite (399 ms for P3HT/UCNPs@SiO_2_ versus 486 ms for UCNPs@SiO_2_) indicated energy transfer from the UCNPs to P3HT, corroborating the RET mechanism.^[^ ^50^, ^51^ ^]^

The RET of the UCNPs is crucial in amplifying the light response and promoting carrier separation. Under NIR illumination, the UCNPs absorb photons and emit high‐energy photons (Figure 4a,b). These emissions are reabsorbed by P3HT, generating additional excitons (Figure 4b). Furthermore, the photogenerated holes diffuse into P3HT, whereas electrons are captured by the lower energy levels of the UCNPs, effectively separating the carriers.^[^ ^52^ ^]^ This dual mechanism boosts the current under NIR. Prolonged programming strengthens the built‐in electric field, enhancing the electron capture capability of UCNPs and suppressing hole‐electron recombination, sustaining current enhancement. Kelvin probe force microscopy (KPFM) was used to gain an in‐depth understanding of the RET‐assisted charge‐trapping mechanism, revealing an increasing surface potential with increasing programming time (Figure 4c–e). In the dark, the average surface potential increases from 79.25 to 713.25 mV as the programming time increases from 0 to 8 s. Additionally, NIR illumination further increases the surface potential owing to electron trapping in UCNPs@SiO_2_ (Figure 4d,e). The observed rise in surface potential difference upon NIR illumination is greater with prolonged programming (Figure 4e,f). Additionally, the surface potential difference (|*φ*_NIR_‐*φ*_dark_|) gradually increases from 7.55, 33.30, and 45.16 to 72.63 mV as the programming time rises. Collectively, these findings support the proposed operational mechanisms.

### 2.5 NIR In‐Sensor RC System for Static and Dynamic Image Recognition

**Figure 5:**
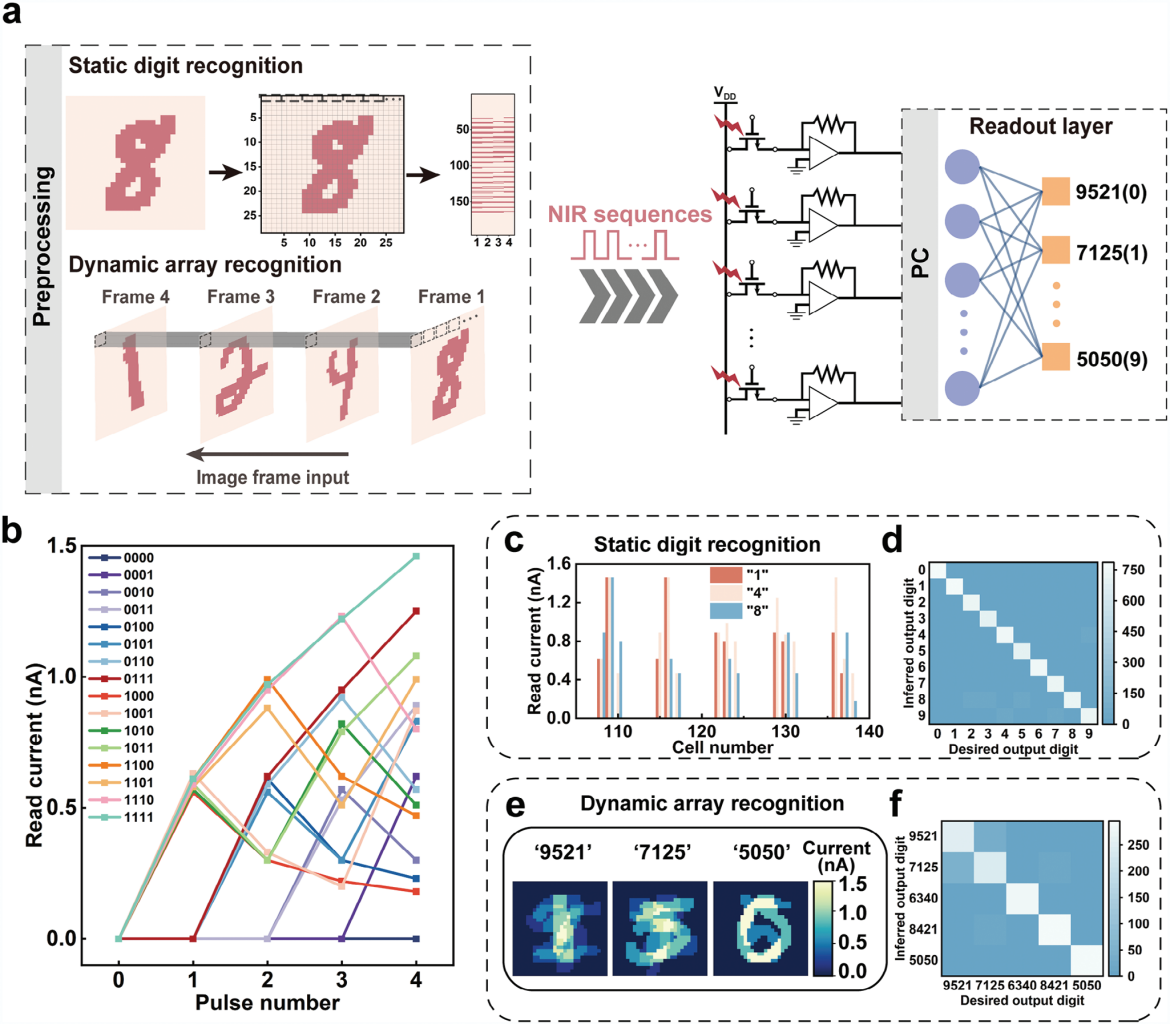
Static/Dynamic handwritten digit recognition tasks using the NIR RC system. a) Scheme of the static handwritten digit/dynamic handwritten array recognition task. Each digit was pre‐segmented into 28 × 28 pixels. The static digit image undergoes a transformation into NIR pulse streams and then fed to the reservoir. The dynamic array in each pixel site was illuminated into the reservoir in a series of frames. The recognition results are generated after inputting the reservoir state into a trained readout function. b) Current responses of the reservoir drained into 16 pulse streams. c) Three examples of reservoir states in the static handwritten digit, presenting significant variations in the reservoir states. d) Confusion matrix revealing the classification results for the experimental and desired outputs. The color depth indicates the extent of such prediction results. e) Three examples of reservoir states in the dynamic handwritten array, presenting reservoir state differences. f) Confusion matrix for dynamic handwritten array recognition. Most arrays remained correctly classified.

The device can function as a reservoir layer in RC systems, and this was further evaluated using static and dynamic handwritten digit recognition tasks (**Figure** **5**a). We used the Mixed National Institute of Standards and Technology (MNIST) database, containing 28 × 28‐pixel images of digits “0” to “9” as input signals for the RC system. Considering the handwritten digit “8,” the original grayscale image was converted into a binary image, enabling each pixel to be represented by “1” or “0” to denote the presence or absence of NIR input. Each row of the 28 pixels was divided into seven 4‐bit pulse sequences, which were sequentially delivered as NIR signals into the RC system using the combined instrument (Figure S4, Supporting Information). The device could distinguish 16 current states induced using different 4‐bit pulse sequences (Figure 5b), allowing the current response of the final bit to serve as a reservoir state. A single device exhibits consistent current states under various NIR pulse cycles (Figure S11, Supporting Information), demonstrating high response uniformity. This facilitates handwritten digit image recognition by training a single‐layer, fully connected neural network readout layer.

Figure 5c presents the ability of the device to distinguish different input patterns, where distinct reservoir states are observed for digits “1,” “4,” and “8.” This underscores the core mechanism of image recognition via feature extraction using RC systems. The system achieved a maximum static recognition accuracy of 91.13% for the MNIST dataset using a single‐layer neural network readout layer (Figure S12a, Supporting Information). Figure 5d presents the corresponding classification confusion matrix.

Dynamic image recognition tasks were executed to further evaluate the capacity of the proposed RC system. Figure 5a reveals that five 4‐digit numbers (“9521,” “7125,” “6340,” “8421,” and “5050”) were loaded into the RC system for recognition and classification. Images are first binarized for dynamic numbers like “8421.” Unlike static image recognition pre‐processing, the dynamic array recognition task considers each pixel as a unit and inputs the NIR‐coded data of each pixel site in the order of 8″ – “4” – “2” – “1, ”which are also included in the MNIST database. For dynamic recognition, each digit within a 4‐digit number was sequentially converted into a 4‐bit NIR pulse sequence matching the 28 × 28 dimensions of the RC system (analogous to the pixel resolution). The system processed these sequences to extract features and generated the corresponding reservoir states, which were fed into a single‐layer neural network for classification. The readout layer achieved a maximum accuracy of 90.07% (Figure S12b, Supporting Information). Figure 5e presents the current values corresponding to three distinct 4‐digit numbers imported into the reservoir. The last two frames were more prominent than the earlier numbers, possibly because of the lower weight assigned to the earlier numbers. The classification confusion matrix (Figure 5f) provides further details on the recognition performance for the dynamic sequences.

### 2.6 Solving a Second‐Order Nonlinear Dynamic Task Using an RC System

RC excels in handling high‐dimensional and nonlinear input data, making it ideal for time‐series systems, such as those in complex dynamics.^[^ ^53^, ^54^ ^]^ Richer reservoir states enhance RC performance, which is traditionally achieved through hardware modifications or continuous voltage adjustments.^[^ ^24^, ^55^, ^56^, ^57^ ^]^ A 1 × 8 array of the UCNPs@SiO_2_/P3HT devices with eight programmed states was illuminated using a single NIR irradiation to obtain high‐dimensional reservoir states from a one‐dimensional input. We can modulate the relaxation time of devices from 2.9 s to 8.9 s, leveraging the interplay between the NIR and programming state (Figures S5b, S6c, Supporting Information), enabling diversity in non‐volatile reservoir states.

**Figure 6:**
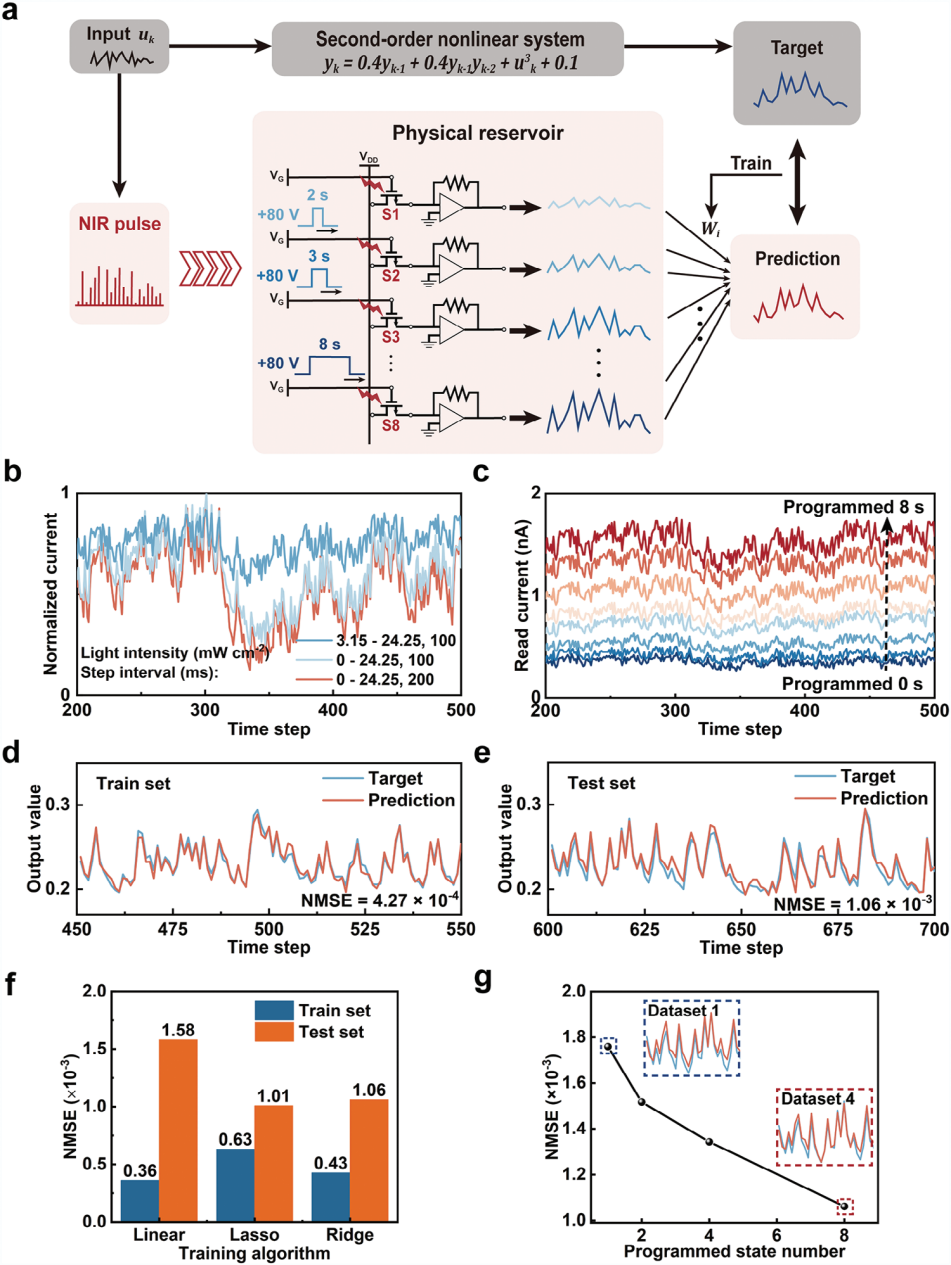
Solving a second‐order nonlinear dynamic equation task. a) The scheme of the task calculated using the UCNPs@SiO_2_‐based reservoir. Inputs (u(k) and 0.5 − u(k)) were applied to devices with various storage states, and the corresponding dynamic response currents were collected for the readout layer. b) The enriched dynamic response currents of the reservoir are derived from varying NIR input pulse parameters. The NIR pulse width of each step in sequence u(k) was fixed at 100 ms. The storage state of the device remained at S8. c) The enriched dynamic response currents of the reservoir derived from varied device states. The NIR pulse width and interval of each step in the sequence u(k) were fixed at 100 ms. d) Target and prediction results of the equation at the training phase. e) Target and prediction results of the equation at the testing phase. f) Comparison of the predictive accuracy of three linear prediction models. g) The relationship between the number of programmed states in the reservoir and the NMSE of the prediction reveals that collecting more response currents with various programmed states leads to a higher prediction accuracy.

We used a second‐order nonlinear dynamic equation task to evaluate the calculation capability of the proposed RC system (**Figure** **6**a). This task involves providing the system with an input signal u(k), within a specified time frame k. The system then generates an output signal y(k) based on a nonlinear transfer function with potential delays. The output signal y(k) is described using the following equation:

(5) $$y\left(k\right)=0.4y\left(k-1\right)+0.4y\left(k-1\right)y\left(k-2\right)+u^{3}\left(k\right)+0.1$$

where k denotes a discrete time, u(k) = * *[0, 0.5] denotes a random input that is converted into an NIR sequence input system for calculation. The output y(k) depends on the current input u(k) and on the outputs y(k−1) and y(k−2) of both previous time frames, obscuring the relationship between y(k) and u(k) (Equation (5)). This dependence aligns with the STM of the RC system, rendering it suitable for these dynamics.

The experimental setup comprised 700 time steps divided into three phases: 50‐step washout, 550‐step training, and 100‐step testing. The proposed device enriches reservoir states by adjusting two variables—the input parameters of the NIR sequence and the programmed state. Thus, the randomly generated time series u(k) and 0.5 − u(k) were linearly mapped to the NIR light intensity range, and the appropriate step time/interval was also selected. Subsequently, both inputs were applied to the device in various programmed states. Figure 6b depicts the ability of the device to modulate its reservoir state via input parameters (light intensity and step time) while maintaining fixed programming. Light intensity variations significantly affected the response trend, whereas step interval changes primarily affected minute current values. In addition, Figure 6c demonstrates that the influence of the programmed state on the reservoir state modulation is independent of the NIR sequence parameters. Increasing programmed time raised the amplitude and relaxation time of the NIR response.

We collected NIR response data from the devices under various programmed states and NIR irradiation parameters to verify the impact of the rich reservoir states on solving the second‐order nonlinear dynamic equation (Tables S1, S2, Supporting Information). Ten virtual nodes were used to represent the reservoir state at each time step to enhance the reservoir state dimensionality (Figure S13, Supporting Information). Dataset 1 served as the foundation and included tests with diverse NIR light intensity ranges and step intervals in a programmed state. Figure S14 (Supporting Information) depicts the target and predicted outputs of the test data in Dataset 1. The agreement between the target and predicted outputs for Dataset 1 resolved the problem. Accurate predictions (NMSE = 1.76 × 10^−3^) are due to the NIR input parameter effectively regulating the states of the physical reservoir. Therefore, Datasets 2, 3, and 4 included test data for 1, 3, and 7 programmed states, respectively, under an NIR input parameter. Figure 6d,e present a drop in NMSE to 4.27 × 10^−4^ (training dataset) and 1.06 × 10^−3^ (test dataset) for Dataset 4 with 8 programmed states. The prediction results achieved using the UCNPs@SiO_2_‐based RC system for the prediction task indicated a superior ability to process temporal information compared with other physical RC systems (Figure S15, Supporting Information). The other two linear prediction models had consistent results and similar prediction accuracies (Figure 6f; Figure S16, Supporting Information). The NMSE progressively decreased with increasing programmed state, which was evident in the prediction accuracy across the datasets (Figure 6d,e,g; Figure S14, Supporting Information). The reservoir size in the dataset was determined using the following equation:

(6) N=10n

where n represents the number of test conditions ($n_{\mathrm{data}\mathrm{set}1}$ = 8, $n_{\mathrm{data}\mathrm{set}2}$ = 10, $n_{\mathrm{data}\mathrm{set}3}$ = 14, and $n_{\mathrm{data}\mathrm{set}4}$ = 22) and constant 10 represents 10 virtual nodes in a time step for each test condition. We fixed the reservoir size at 80 by adjusting the virtual nodes and used an 80‐dimensional readout layer to isolate the direct impact of programmed states on the accuracy. Despite the fixed size, the prediction accuracy of the test set improved with an increasing number of programmed states (Figure S17, Supporting Information). The NMSE decreased from 1.76 × 10^−3^ (one state) to 1.14 × 10^−3^ (eight states), highlighting the effectiveness of using multiple programmed states to enrich reservoir states and achieve better accuracy in solving second‐order non‐linear dynamic equations. Highlighting the advantages of UCNPs@SiO_2_‐based devices in RC, we compared the optoelectronic performance of the device, reservoir state richness, and RC accuracy with other reports; Table S3 (Supporting Information) details the results. Our device exhibited abundant optoelectronic response behavior, rich reservoir regulation terminals, and high computing power, indicating its potential for future in‐sensor RC systems.

## 3 Conclusion

We present a simple‐structured NIR retinomorphic device that can perceive and encode NIR information (≈980 nm) to extend human sensation beyond the visible spectra. This three‐terminal transistor was designed by spin‐coating the UCNPs@SiO_2_/P3HT composite onto a substrate with a four‐in‐one effect, including a semiconductor channel, light‐sensitive layer, charge‐trapping layer, and tunneling dielectric layer. The strong absorption band of the p‐type P3HT (400–700 nm) overlapped with the dominant UCNPs@SiO_2_ emission peak (≈545 nm), thereby promoting efficient RET. UCNPs@SiO_2_ nanostructures were used in this study, and the SiO_2_ shell was self‐assembled onto the UCNP core during the synthesis to function as a tunneling dielectric layer, supplying a suitable well for photoinduced charge trapping. Devices with different electrically programmed states vary in synaptic response under NIR irradiance, which is crucial for achieving high dimensionality— a desirable characteristic for RC. Beyond light perception and encoding, the device possesses multilevel data storage capability (≥8 levels) with exceptional stability (≥2000 s) and durability (≥100 cycles) by manipulating the programming time in the dark. This facilitates potential applications in non‐volatile memory devices. The device was successfully implemented as a high‐dimensional reservoir for RC tasks. The system demonstrated remarkable accuracy in recognizing handwritten digits in the static (91.13%) and dynamic (90.07%) recognition modes, demonstrating its robust performance in complex information processing. The device tackles intricate computations like solving second‐order nonlinear dynamic equations with minimal errors (NMSE of 1.06 × 10⁻^3^ during prediction). These findings highlight the potential of NIR in‐sensor RC systems as powerful tools for future computations and predictions in sequential tasks, offering a compelling alternative to conventional computing paradigms.

## 4 Experimental Section

### Device Fabrication

The UCNPs@SiO_2_‐based device had a bottom‐gate top‐contact structure. A pre‐cleaned silicon wafer with 300‐nm SiO_2_ was used as the substrate. P3HT (15 mg) (305063, Xi'an Yuri Solar Co. Ltd) was dissolved in 3 ml of methylbenzene (Sigma Aldrich), and the solution was filtered after heating at 80 °C for 20 min. The solvent ethanol used to store NaYF_4_:Yb, Er/SiO_2_ (XF193‐4, XFNANO Materials Co. Ltd) was evaporated at 125 °C, then mixed with the P3HT solution to attain a uniform concentration of 5 mg ml^−1^. Ultrasonication was performed for 30 min to enhance the stability of the UCNPs@SiO_2_ dispersion. The sonicated P3HT/UCNPs@SiO_2_ solution was applied onto the substrates via spin‐coating at 1500 rpm for 30 s, subsequently undergoing thermal treatment at 100 °C for 20 min to complete the process. Finally, 50 nm Au source and drain electrodes were thermally evaporated (rate: 0.1 Å s^−1^) on the film via a shadow mask under 4 × 10^−4^ Pa, with an as‐prepared flash memory channel length of 50 µm. Flexible devices were fabricated on a polyethylene terephthalate (PET) substrate (200 µm thickness, South China Science and Technology Co. Ltd) with 185‐nm indium tin oxide. A 50‐nm‐thick layer of Al_2_O_3_ was formed as the gate dielectric, using an atomic layer deposition system while maintaining a substrate temperature of 80 °C. The other processes were the same as those used for the rigid devices. All the above operations were performed in a glovebox under a nitrogen atmosphere.

### Electrical/Optical Measurements

The electrical properties of the UCNPs@SiO_2_‐based device, including *I*–*V* characteristics, *I*–*T* characteristics, and dynamic behavior under electrical/optical pulse stimulation, were assessed using a probe station integrated with the Keithley B2902A precision source/measure system. A 980‐nm laser (Laserwave) and signal generator (Gwinstel AFG‐2225) were combined for the optical measurements. Figure S4 (Supporting Information) illustrates the equipment connections. The power density of NIR was measured using a power meter (Thorlabs S121C). All the above tests were performed in a glovebox under a nitrogen atmosphere at room temperature.

### Material Characterization

A cross‐sectional image of the device was characterized using a field emission SEM (FEI Scios, Thermo Fisher). The morphologies and potentials of the film surfaces were observed using AFM and KPFM (Bruker). The size distribution of UCNPs@SiO_2_ was characterized using the TEM (Titan Cubed Themis G2300, FEI). The absorption spectra were recorded using a UV–vis spectrophotometer (Cary60, Agilent). The PL spectra and decay curves were obtained using a steady‐state/lifetime spectrofluorometer (FLS‐1000, Edinburgh) equipped with a 980‐nm laser as the excitation source.

### Static/Dynamic Image Recognition

The device was only in the storage state S8 performed the task. The MNIST dataset provided all the images used in the image recognition tasks. Each 28 × 28 image pixel is converted to a binary state—“0” or “1”—via a NIR signal input to the device. The light intensity of “1” and “0” were 24.25 mW cm^−2^ and 0 mW cm^−2^, respectively. The pulse width of the NIR signal of each pixel was 0.5 s, and the pulse interval was 0.1 s. At the same time, the response current was measured by applying constant drain voltage (*V*_DS_ = –5 V) and gate voltage (*V*_G_ = –10 V) to the UCNPs@SiO_2_‐based device. The NIR image pre‐processing was based on previous reports.^[^ ^53^ ^]^

Weight training was performed in Python using the softmax activation function. The cross‐entropy loss function was defined to determine the discrepancy between the predicted and target values, and gradient descent was used to iterate the weight of the readout layer. Static images had 40 000 training and testing samples; 80% of the dataset was used as the training set (32 000), and 20% was used as the test set (8000). Conversely, dynamic arrays had 4000 for training and testing samples, with 70% (2800) and 30% (1200) used as the training and test sets, respectively. The learning rates for the static and dynamic recognition were 0.001 and 0.003, respectively.

### Solving Second‐Order Nonlinear Dynamic Equation Task

Figure S4 (Supporting Information) presents the workflow for NIR pulse stream generation. First, the sequences u(k) and 0.5 − u(k) were randomly generated and converted into a voltage signal sequence of equal proportions via a signal generator. Subsequently, the voltage signal sequence pulses control the 980‐nm laser output NIR pulse stream to the device. The width/interval of the NIR pulse and the light intensity range can be predefined by adjusting the voltage signal parameters. The *I*_DS_ response of the UCNPs@SiO_2_‐based device was measured under constant drain voltage (*V*_DS_ = −10 V) and gate voltage (*V*_G_ = −10 V), and ten virtual nodes were obtained from each testing condition.

The training of the readout layer was based on the literature with a slight modification.^[^ ^53^, ^56^ ^]^. The training and prediction tasks were completed using Python. The reservoir states $X_{i}\left(k\right)$ were obtained from the response current, and the reservoir output $y_{p}\left(k\right)$ was the linear combination of $X_{i}\left(k\right)$ and the readout weights $w_{i}$ as follows:

(7) $$y_{p}\;\left(k\right)=\sum_{i=1}^{N}w_{i}X_{i}+b$$

where N and b represent the reservoir size and bias, respectively. Regarding the weight training of the readout layer, the study primarily used ridge regression in addition to linear and lasso regressions for comparison. The cost functions $J_{\mathrm{ridge}}\left(W\right)$, $J_{\mathrm{line}\mathrm{ar}}\left(W\right)$, and $J_{\mathrm{lasso}}\left(W\right)$ in the ridge regression are defined as follows:

(8) $$J_{ridge}=\frac{1}{2T}\;\sum_{k=1}^{T}{\left[y_{p}\left(k\right)-y\left(k\right)\right]}^{2}+\lambda \sum_{i=0}^{N}{w_{i}}^{2}$$

(9) $$J_{linear}\;\left(W\right)=\frac{1}{2T}\;\sum_{k=1}^{T}{\left[y_{p}\left(k\right)-y\left(k\right)\right]}^{2}$$

(10) $$J_{lasso}\;\left(W\right)=\frac{1}{2T}\;\sum_{k=1}^{T}{\left[y_{p}\left(k\right)-y\left(k\right)\right]}^{2}+\lambda \sum_{i=0}^{N}\left|w_{i}\right|$$

where T, λ, and y(k) are the data length in the training phase, regularization parameter, and desired output, respectively. T = 550 was fixed for all the tasks, and $\lambda_{\mathrm{lasso}}$ was set to 2 × 10^−5^ for lasso regression. Table S4 (Supporting Information) details the $\lambda_{\mathrm{ridge}}$ values for ridge regression.

Upon acquiring the readout weights, the computational performance was assessed using NMSE to solve the second‐order nonlinear dynamic equation assignment. The NMSE is defined as follows:

(11) $$NMSE=\frac{\sum_{k=1}^{T}{\left[y_{p}\left(k\right)-y\left(k\right)\right]}^{2}}{\sum_{k=1}^{T}y^{2}\left(k\right)}\;$$

where T is the data length in the training phase (T = 550) or the test phase (T = 100).

## Conflict of Interest

The authors declare no conflict of interest.

## Supporting information



Supporting Information

## Data Availability

The data that support the findings of this study are available from the corresponding author upon reasonable request.
