# Supplementary material for: A Near‐Infrared Retinomorphic Device with High Dimensionality Reservoir Expression
Source: Adv Mater. 2024 Oct 10;36(48):2411225. doi: 10.1002/adma.202411225 (PMC11602693; doi:10.1002/adma.202411225)
Supplement: Supplementary file 1 — Supporting Information [file ADMA-36-2411225-s001.docx]

Supporting Information

A Near-Infrared Retinomorphic Device with High Dimensionality Reservoir Expression

Yan-Bing Leng, Ziyu Lv*, Shengming Huang, Peng Xie, Hua-Xin Li, Shirui Zhu, Tao Sun, You Zhou, Yongbiao Zhai, Qingxiu Li, Guanglong Ding, Ye Zhou, and Su-Ting Han*

Y.-B. Leng, S. Zhu, S.-T. Han

Department of Applied Biology and Chemical Technology

The Hong Kong Polytechnic University

Kowloon, Hong Kong 999077, P. R. China
E-mail: suting.han@polyu.edu.hk

Z. Lv, S. Huang, H.-X. Li, Y. Zhai

College of Electronics and Information Engineering

Shenzhen University

Shenzhen 518060, P. R. China

E-mail: lvziyu@szu.edu.cn

P. Xie, T. Sun, Y. Zhou, Q. Li

Institute of Microscale Optoelectronics

Shenzhen University

Shenzhen 518060, P. R. China

G. Ding, Y. Zhou

Institute for Advanced Study

Shenzhen University

Shenzhen 518060, P. R. China


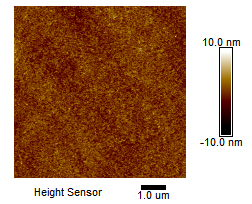


**Figure S1.** The morphological characterization of pristine P3HT film. AFM image of P3HT film surface with ~0.939 nm average roughness, indicates the surface of P3HT is uniform and smooth.


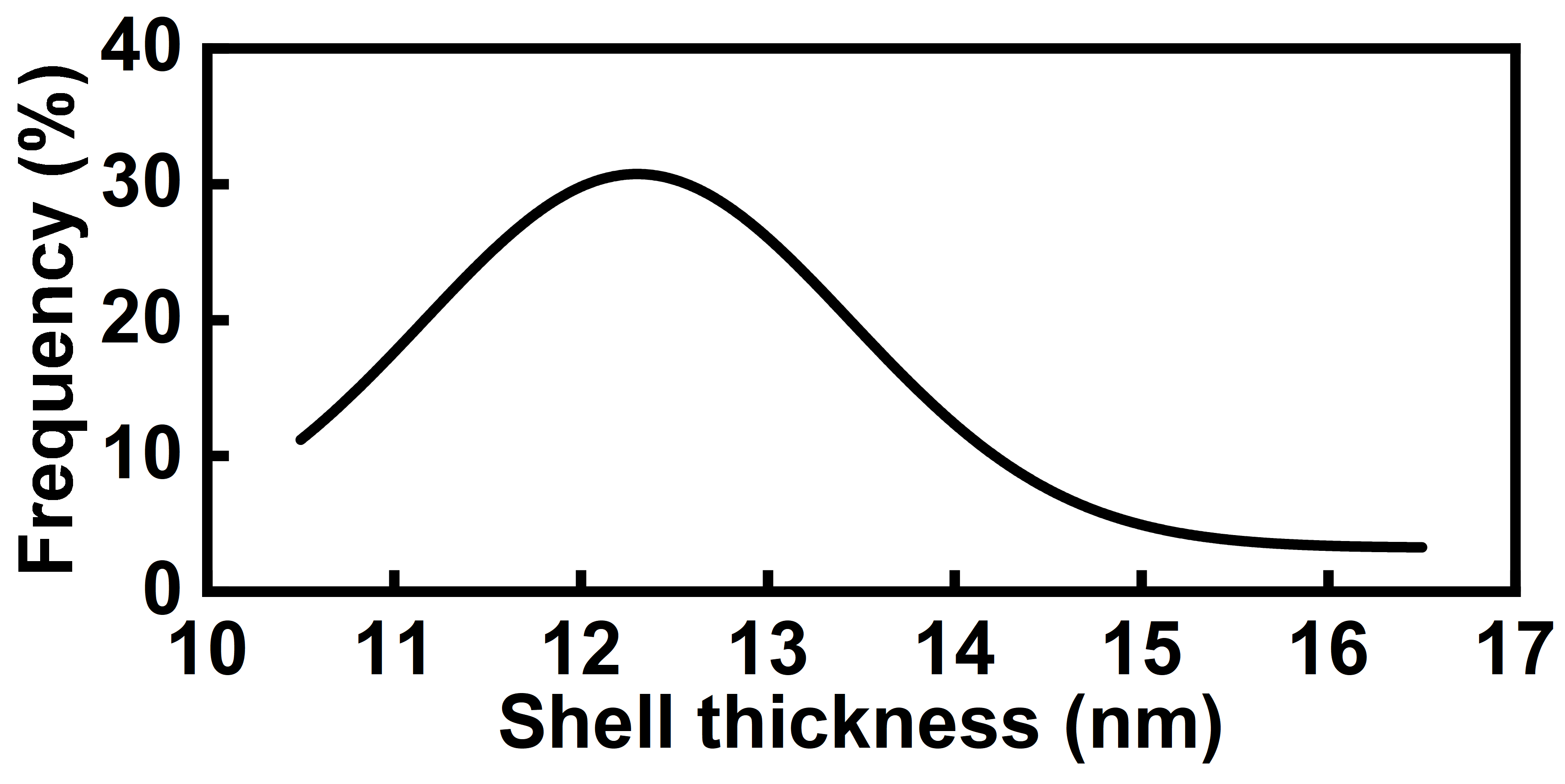


**Figure S2.** Gaussian statistics of SiO_2_ shell thickness distribution of UCNPs@SiO_2_ in TEM image. The shell thickness of 20 complete UCNPs@SiO_2_ samples have been calculated. The average SiO_2_ shell thickness is ~12.48 nm, and the distribution data can be well fitted by Gaussian statistical function.


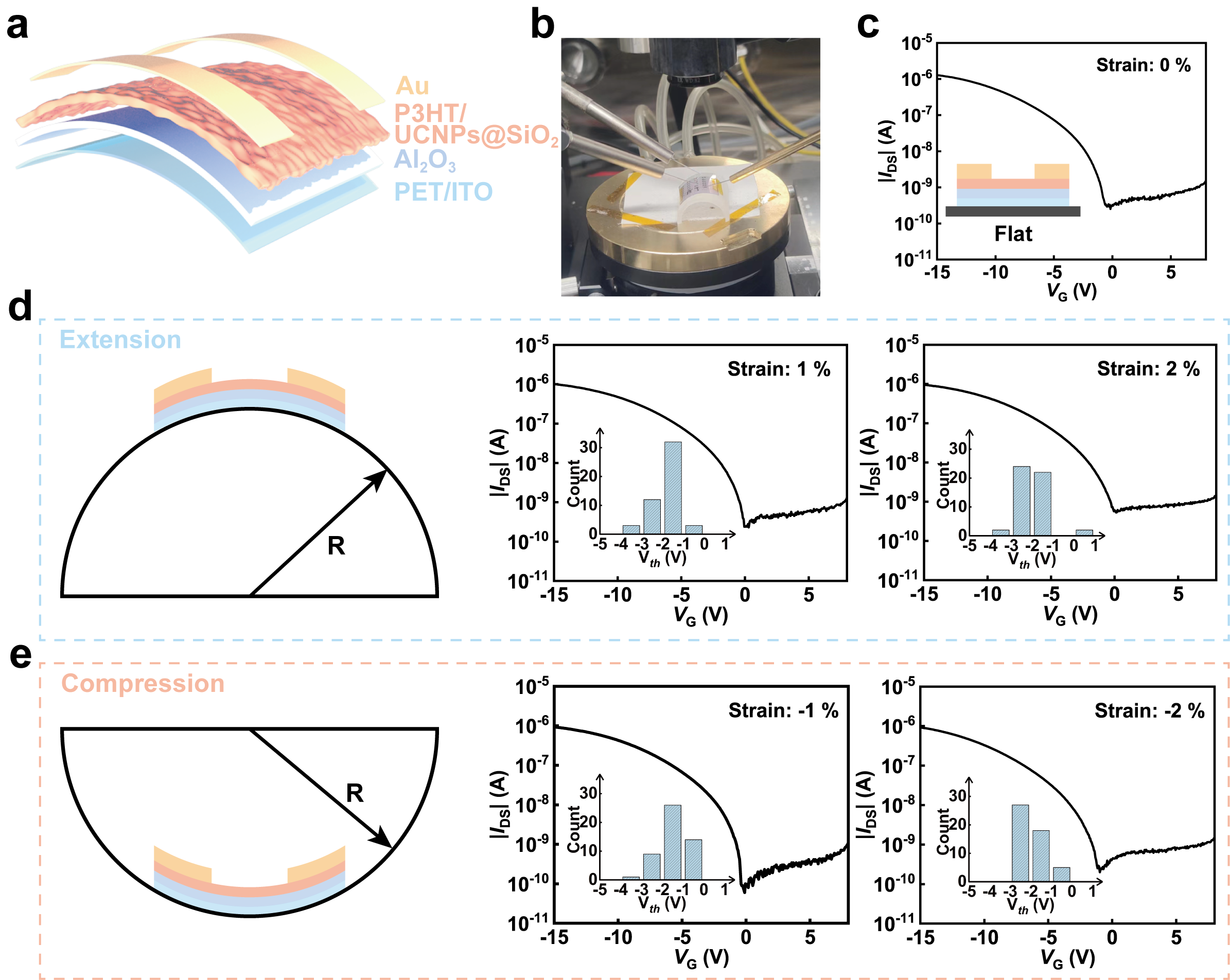


**Figure S3.** Implementation and characteristics of flexible P3HT/UCNPs@SiO_2_ device. a) Schematic of the P3HT/UCNPs@SiO_2_ device fabricated on a flexible polyethylene terephthalate (PET) substrate. The detailed fabrication process can be found in the Methods. b) Photograph of the bending test. c) The transfer curve of flexible device without strain conditions (*V*_DS_ = –10 V). d) Schematic illustration and testing of the flexible device under tensile strain conditions (strain: 1%, 2%). Inset is the *V*_th_ distribution of 50 devices under each tensile strain condition. The strain is extracted by strain = *D*/2*R*, where *D* is the thickness of the PET film (0.2 mm), and *R* is the bending radius.^[1]^ e) Schematic illustration and testing of the flexible device under compressive strain conditions (strain: –1%, –2%). Inset is the *V*_th_ distribution of 50 devices under each tensile strain condition.


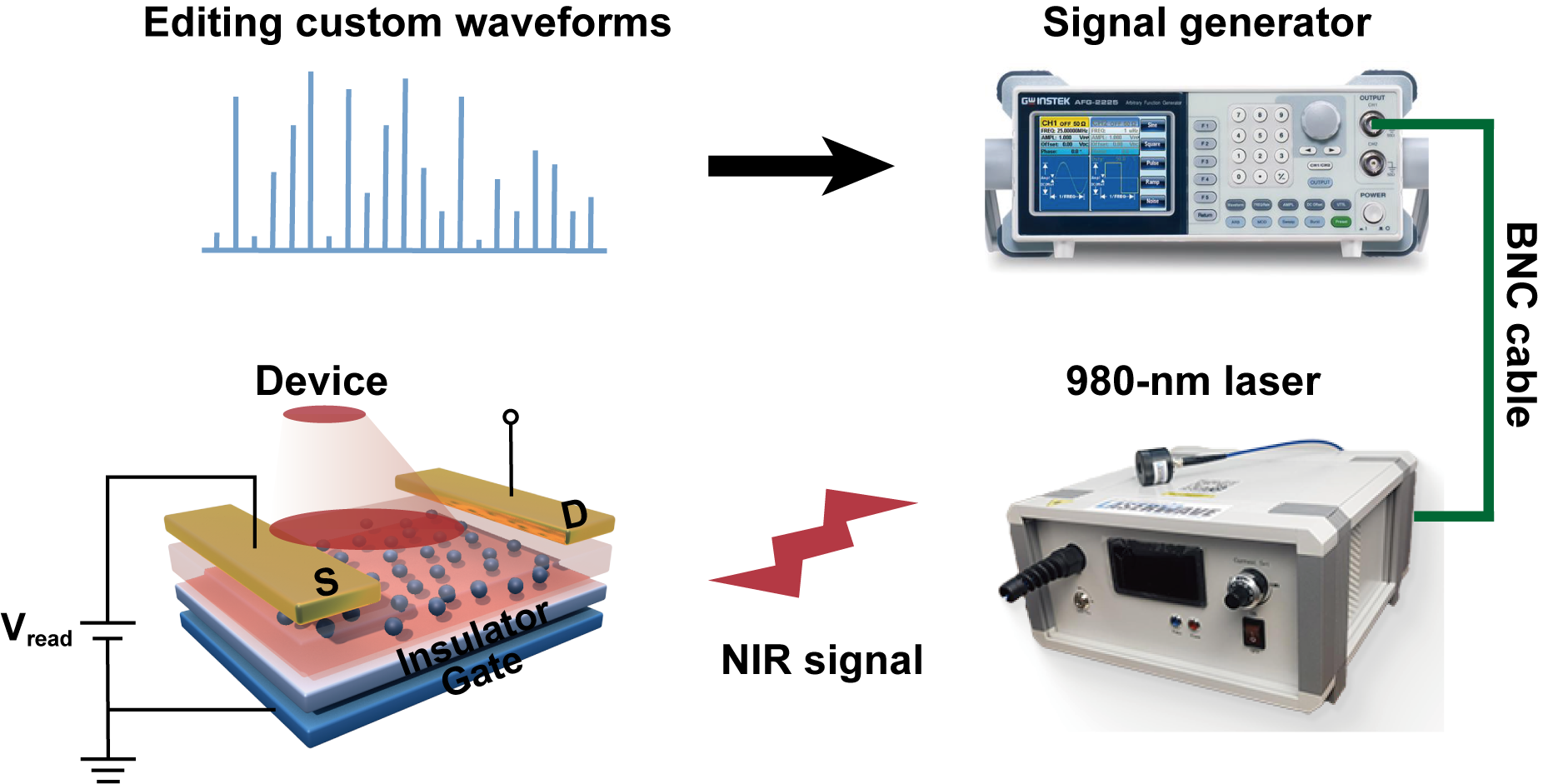


**Figure S4.** Schematic flow diagram for accurate testing of device’s NIR optoelectronic characteristics. Initially, the edited custom waveforms are imported into signal generator, which then transmits the resulting electrical control signal to 980-nm laser via BNC cable. The laser subsequently emits NIR signals characterized by precise pulse width, interval, and intensity in response to the electrical control signal. In conclusion, the semiconductor analyzer is employed to measure device’s current state accurately. This test method was used in all optical response tests of device in this paper.


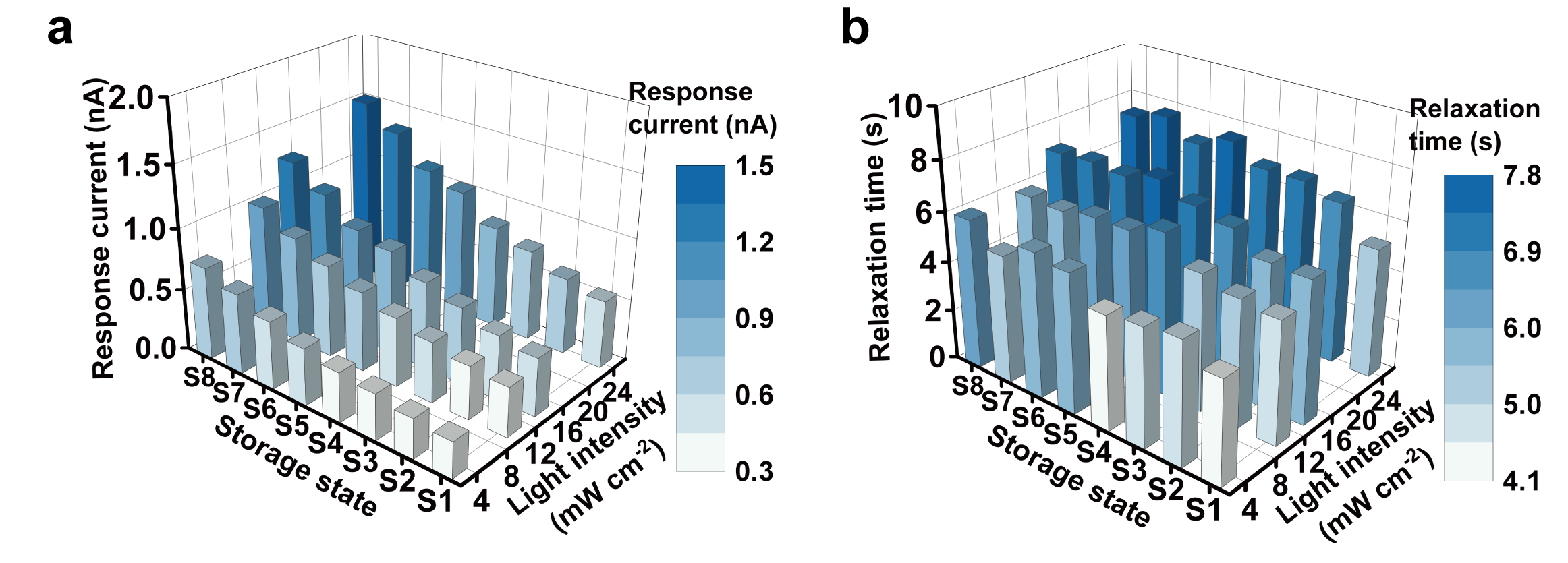


**Figure S5.** Optoelectronic characteristics of devices with different NIR light intensities. a) The distribution of response current of device after applying various light intensities. Optical response current is modulated by both storage state and light intensity. b) The distribution of relaxation time of device after applying various light intensities. Both the longer programming time and the higher light intensity can make device achieve the longer relaxation time.


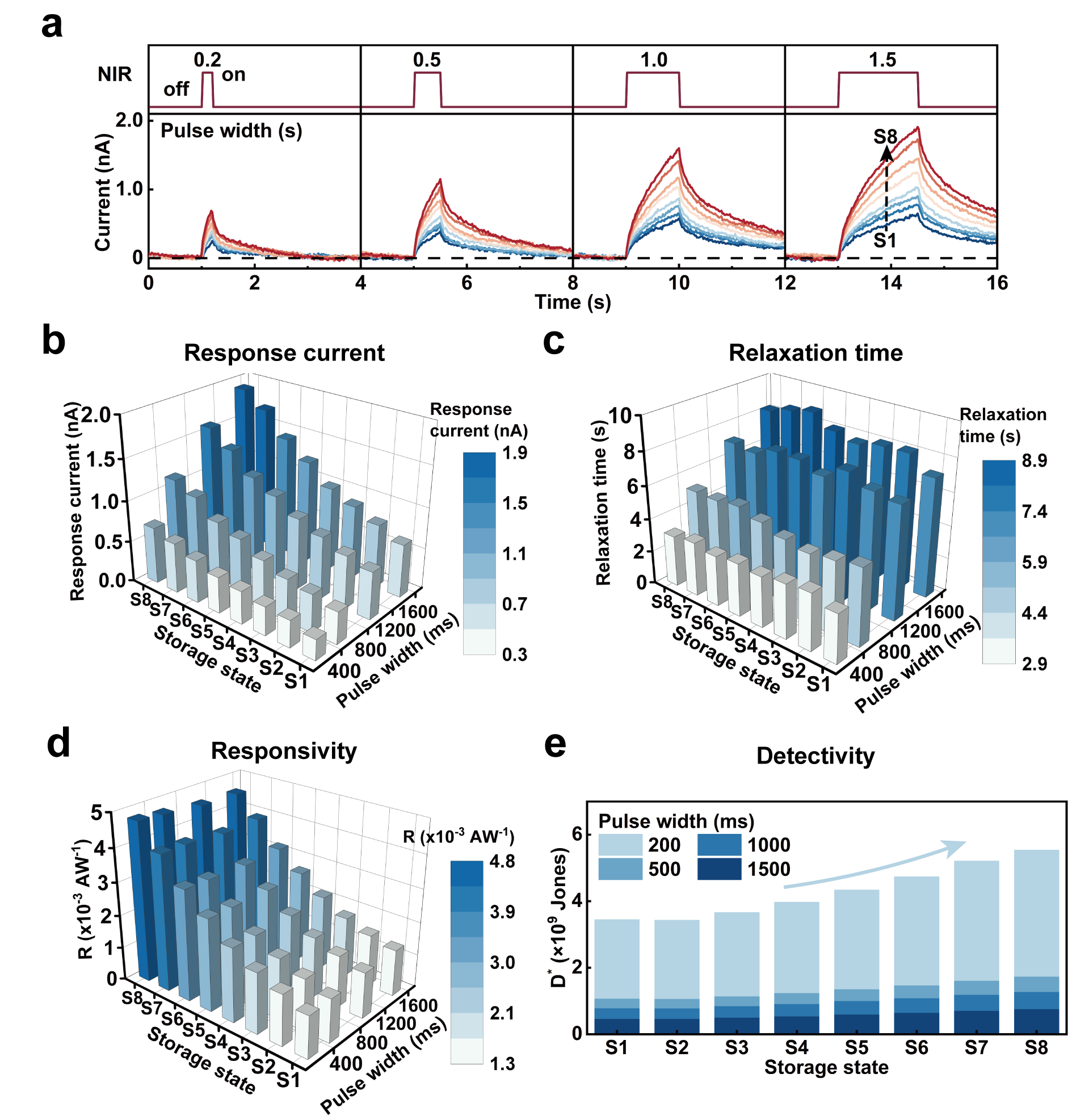


**Figure S6.** Optoelectronic characteristics of devices with different NIR pulse widths. a) The current response following the application of NIR light pulse of varying pulse widths (200, 500, 1000 and 1500 ms, 24.25 mW cm^–2^ pulses) under various storage states. b) The distribution of response current of device after applying various pulse widths. Optical response current is modulated by both storage state and pulse widths. c) The distribution of relaxation time of device after applying various pulse widths. The modulation effect of pulse width on relaxation time is more obvious than that of storage state. d) *R* characteristics of the distinct storage state under different NIR pulse width. The introduction of additional storage states can significantly adjust responsivity to NIR light with various pulse widths. e) *D^*^* characteristics of the distinct storage state under different NIR pulse width. Storage state with longer programming time leads to the higher NIR detectivity, especially at relatively short light pulse width.


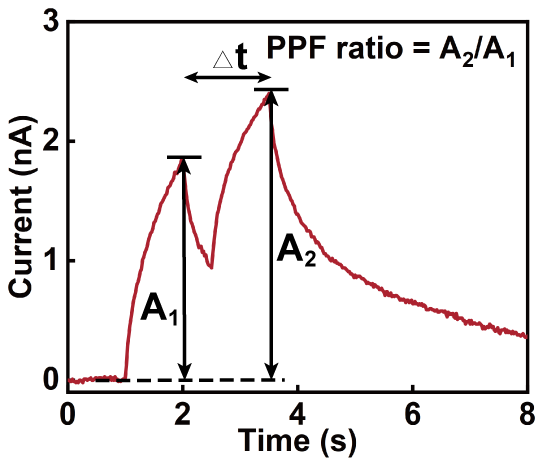


**Figure S7.** The light-induced PPF behavior under 980 nm illumination. The light-induced PPF behavior of the UCNPs@SiO_2_-based device and the definition of *A*_1_, *A*_2_, the time interval △*t* and the calculated formula of PPF ratio.


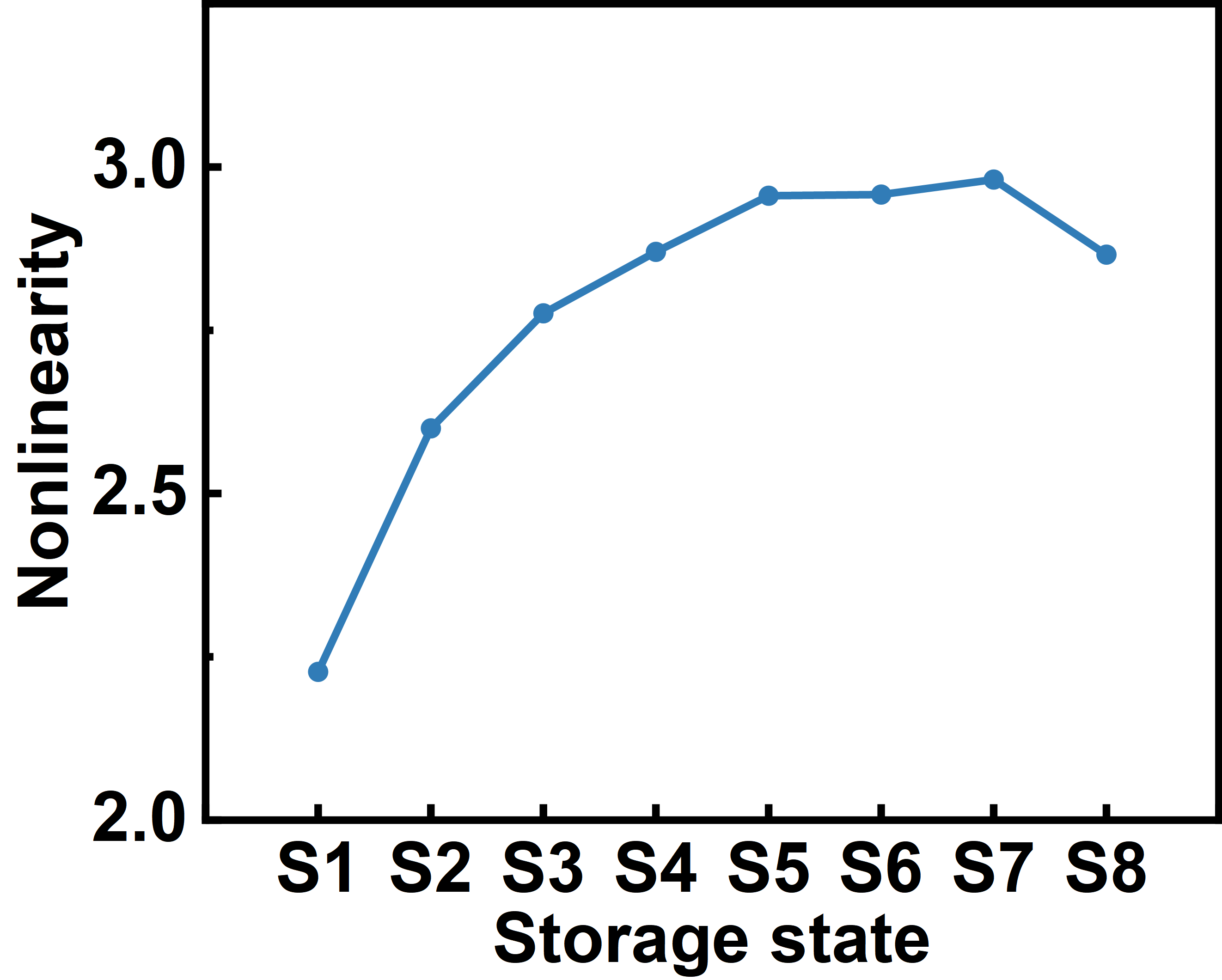


**Figure S8.** Variation trend between response nonlinearity and storage state of device. Storage state with longer programming time expands the range of response current while sacrificing overall linearity.


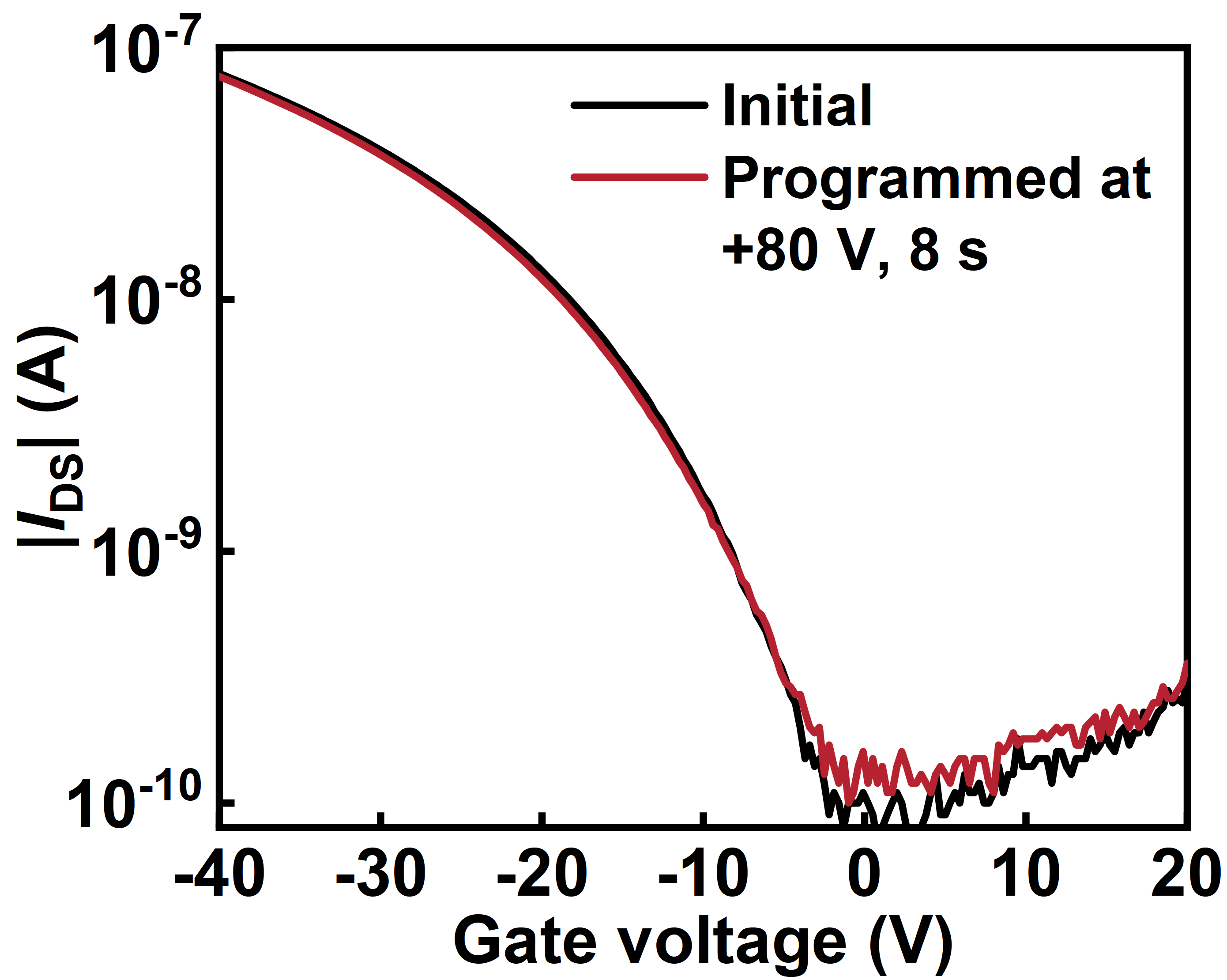


**Figure S9.** Transfer characteristics of the pristine P3HT devices under program operation. Following an 8 s programming time at a voltage of +80 V, the transfer characteristic curve of pristine P3HT device is measured. The finding demonstrate that storage state of device does not change significantly compare to initial state, indicating SiO_2_-coated UCNPs play a leading role in the transformation of storage state.


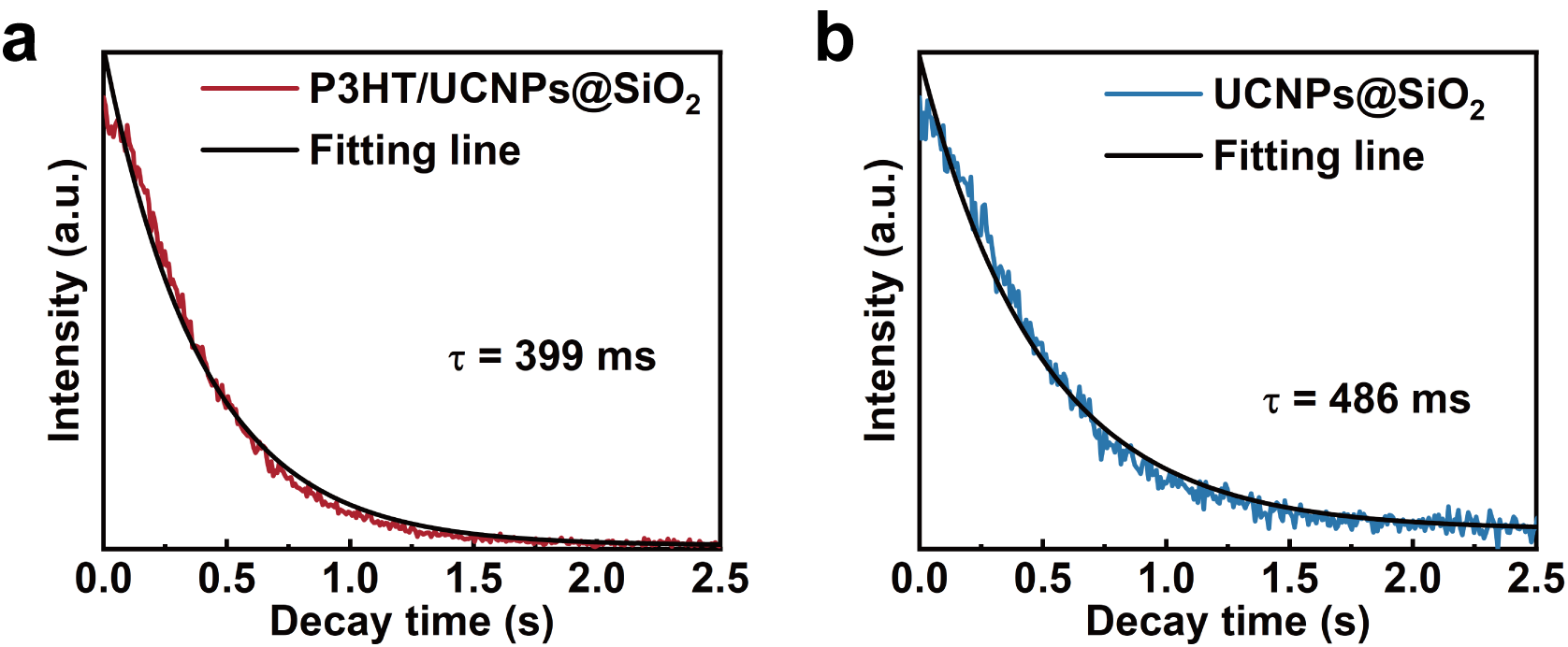


**Figure S10.** Photoluminescence decay curves of a) P3HT/ UCNPs@SiO_2_ layer and b) UCNPs@SiO_2_ layer. The fluorescence lifetime of P3HT/ UCNPs@SiO_2_ layer is shorter than that of UCNPs@SiO_2_ layer, which is caused by energy transfer occurring inside the upconversion material.^[2,3]^


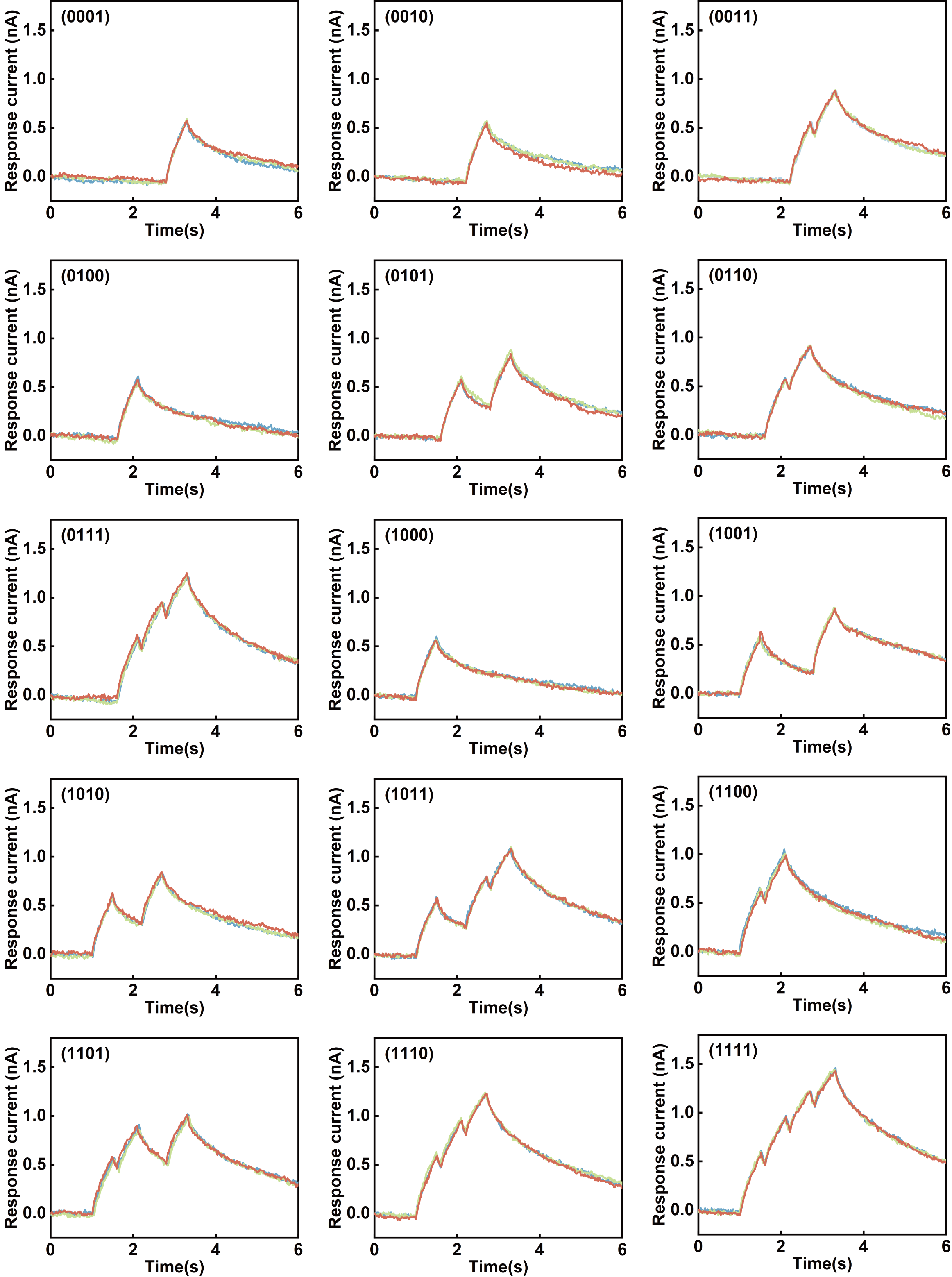


**Figure S11.** Current response from a single device to 15 different pulse stimulations. 3 cycles were tested in this plot (red line, green line and blue line). The device displays similar current variation and relaxation level to each pulse stimulations.


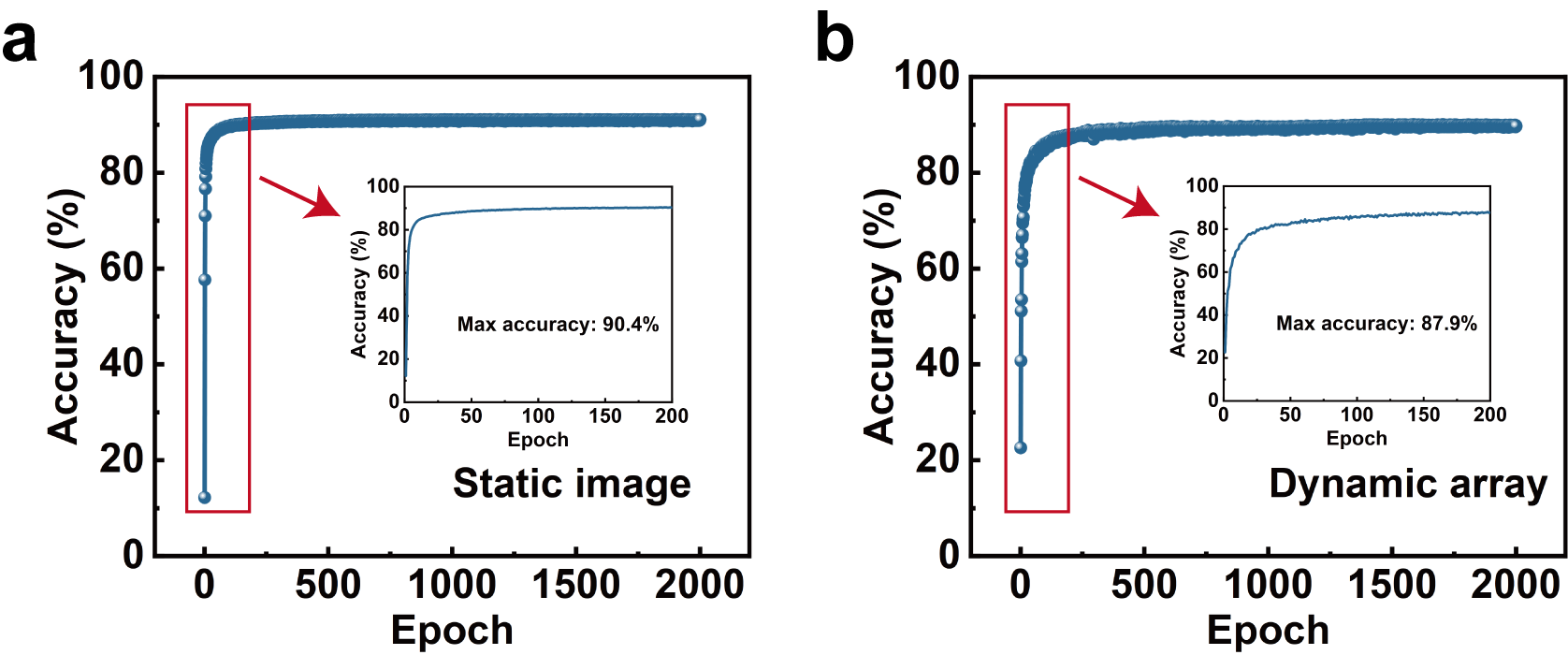


**Figure S12.** Accuracy of a) static image and b) dynamic array recognition during 2000 training epochs. During the 2000 training epochs, recognition accuracy of static image is up to 91.13%, and recognition accuracy of dynamic array is up to 90.07%.


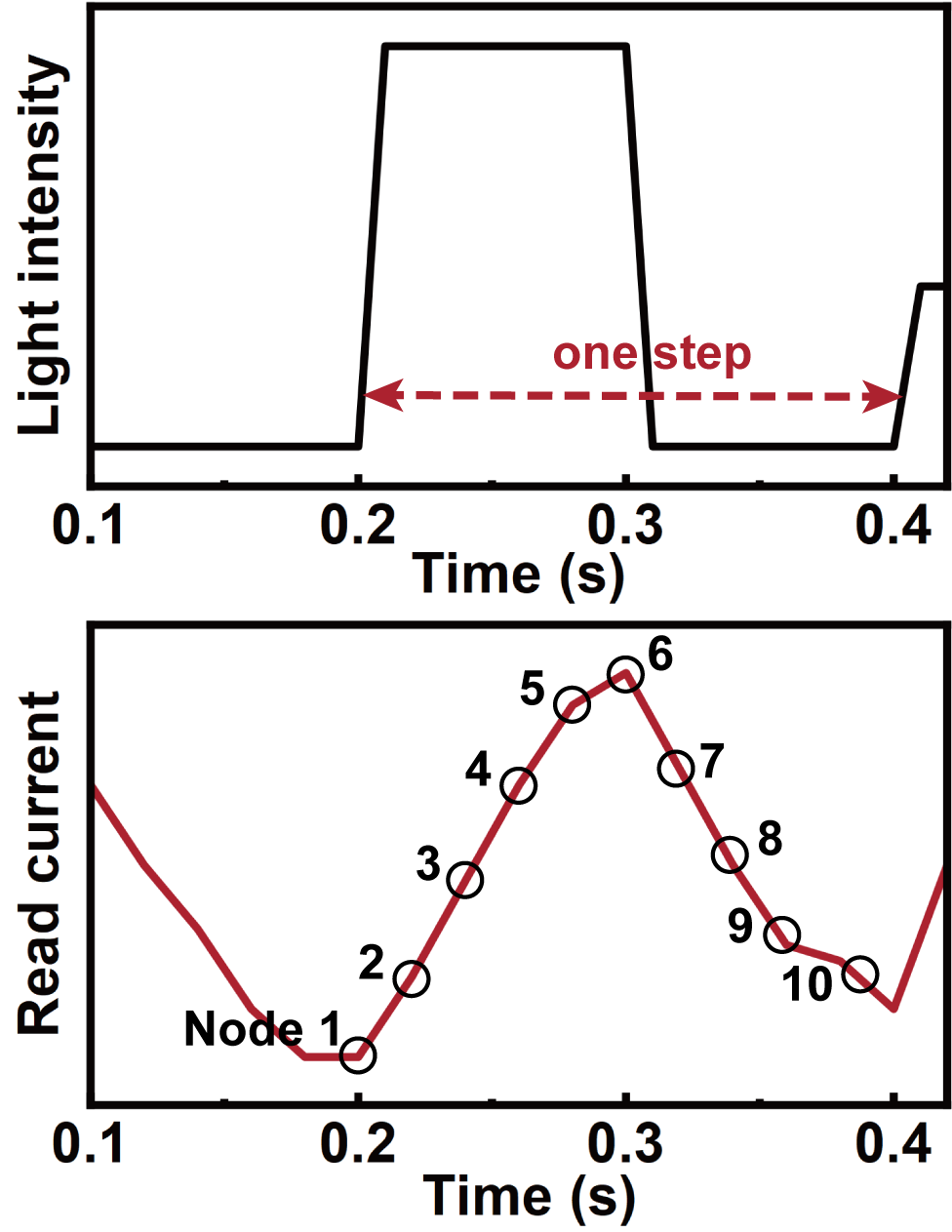


**Figure S13.** Diagram of virtual node. The method for obtaining 10 virtual nodes in one step, with an interval of 20 ms between adjacent virtual nodes.

**
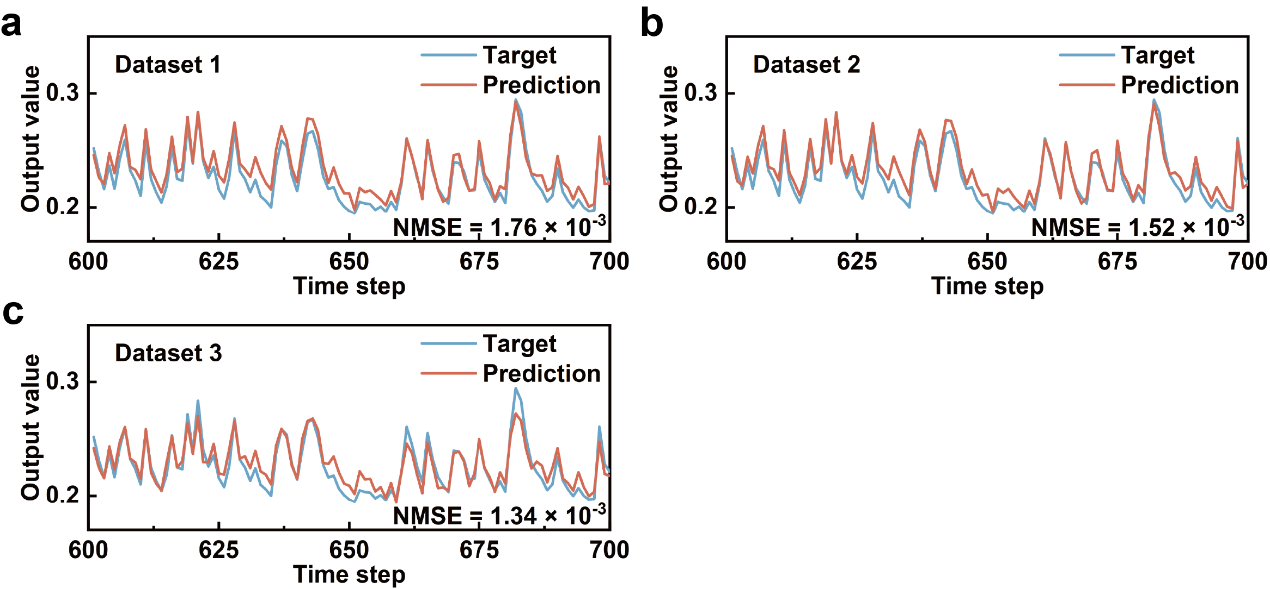
**

**Figure S14.** Prediction of a) dataset 1, b) dataset 2 and c) dataset 3 for test set (600 – 700 time step) with unequal reservoir size. The prediction of dataset 4 is shown in Fig. 5d and 5e of the passage. The findings show that prediction accuracy increases with the rise of the number of storage states in the reservoir.


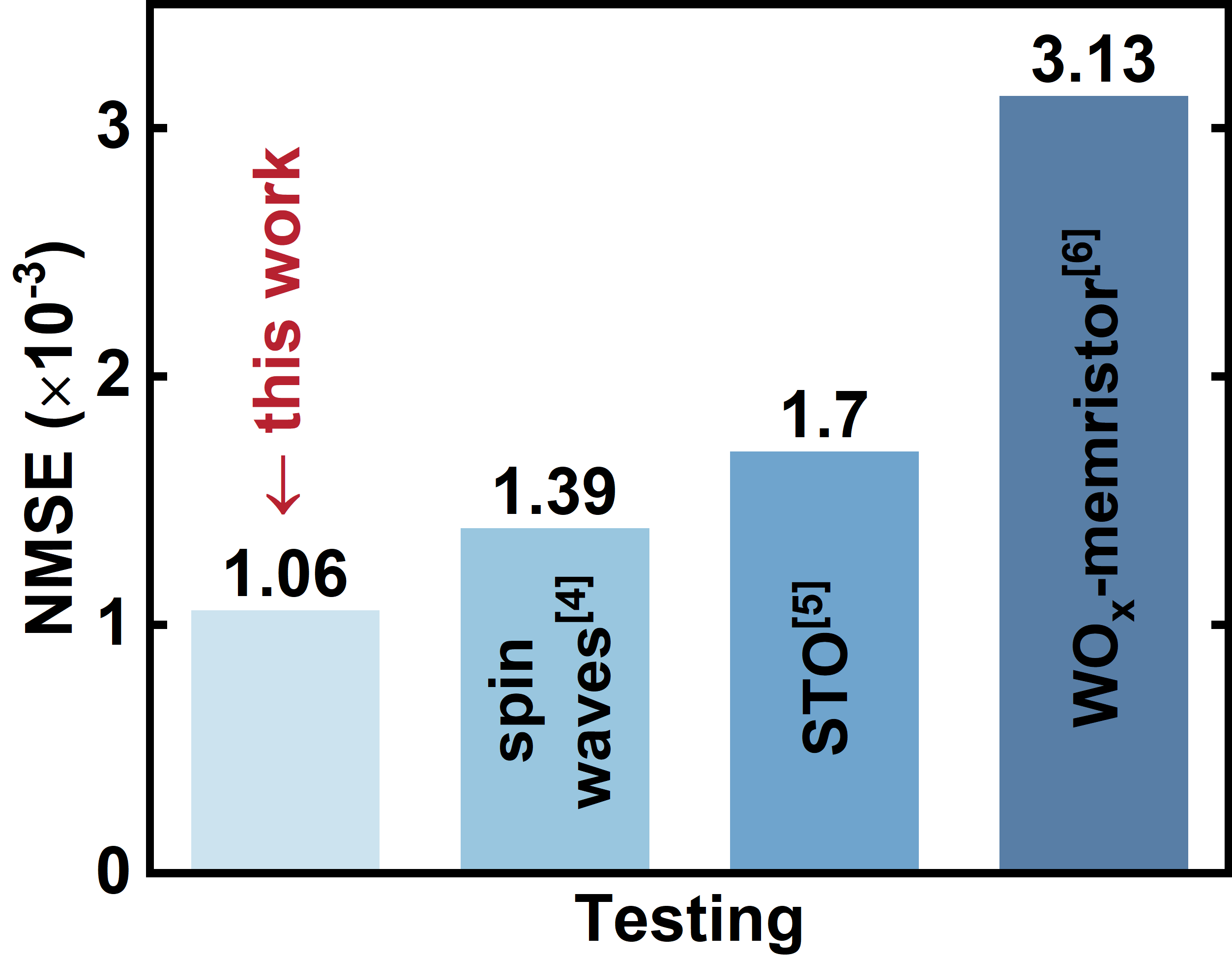


**Figure S15.** NMSE compared to other physical reservoirs.


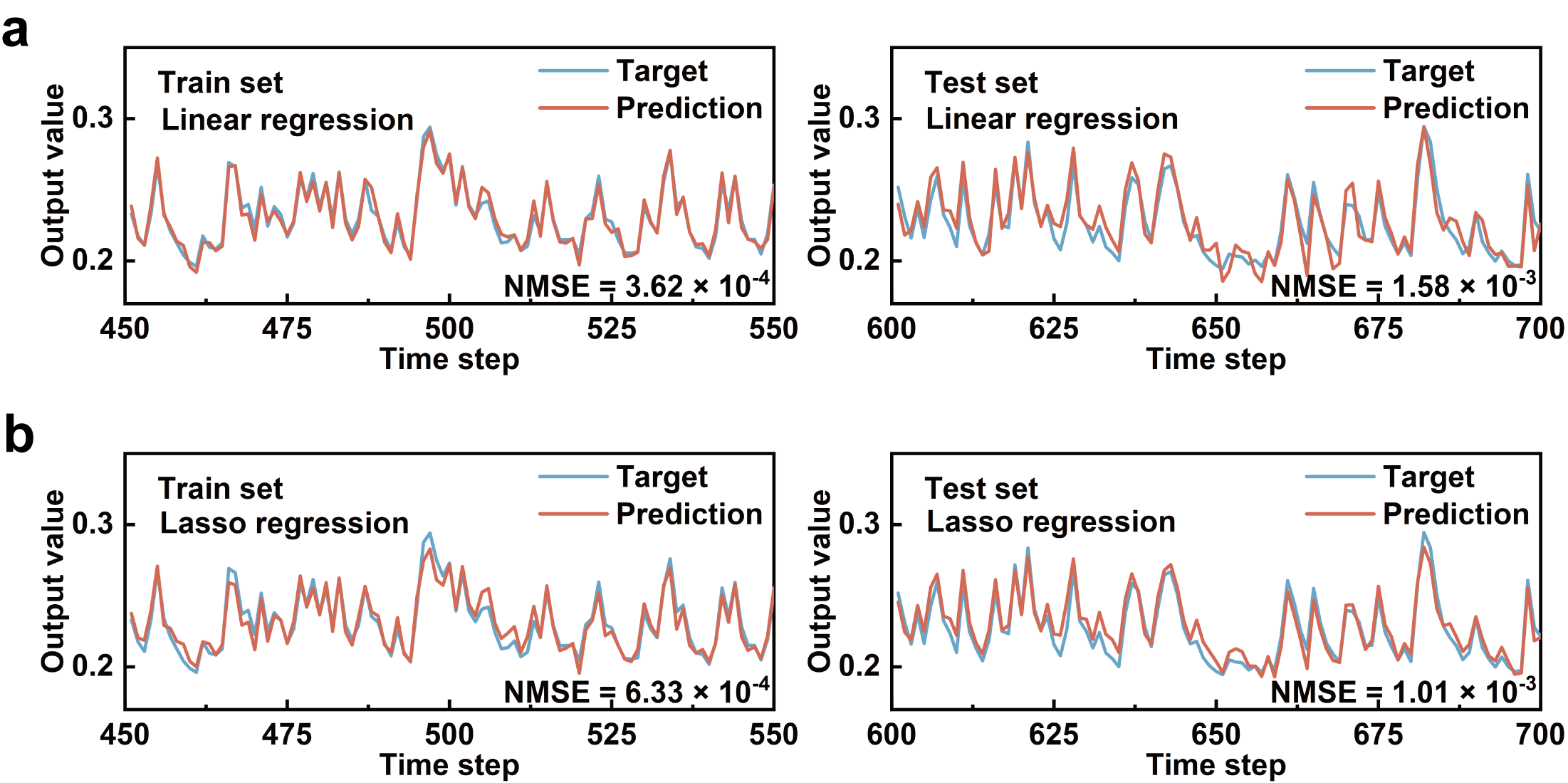


**Figure S16.** Prediction of dataset 4 under a) linear regression and b) lasso regression model. Both linear regression and lasso regression model can make RC system have excellent prediction performance similar to that of ridge regression model.

**
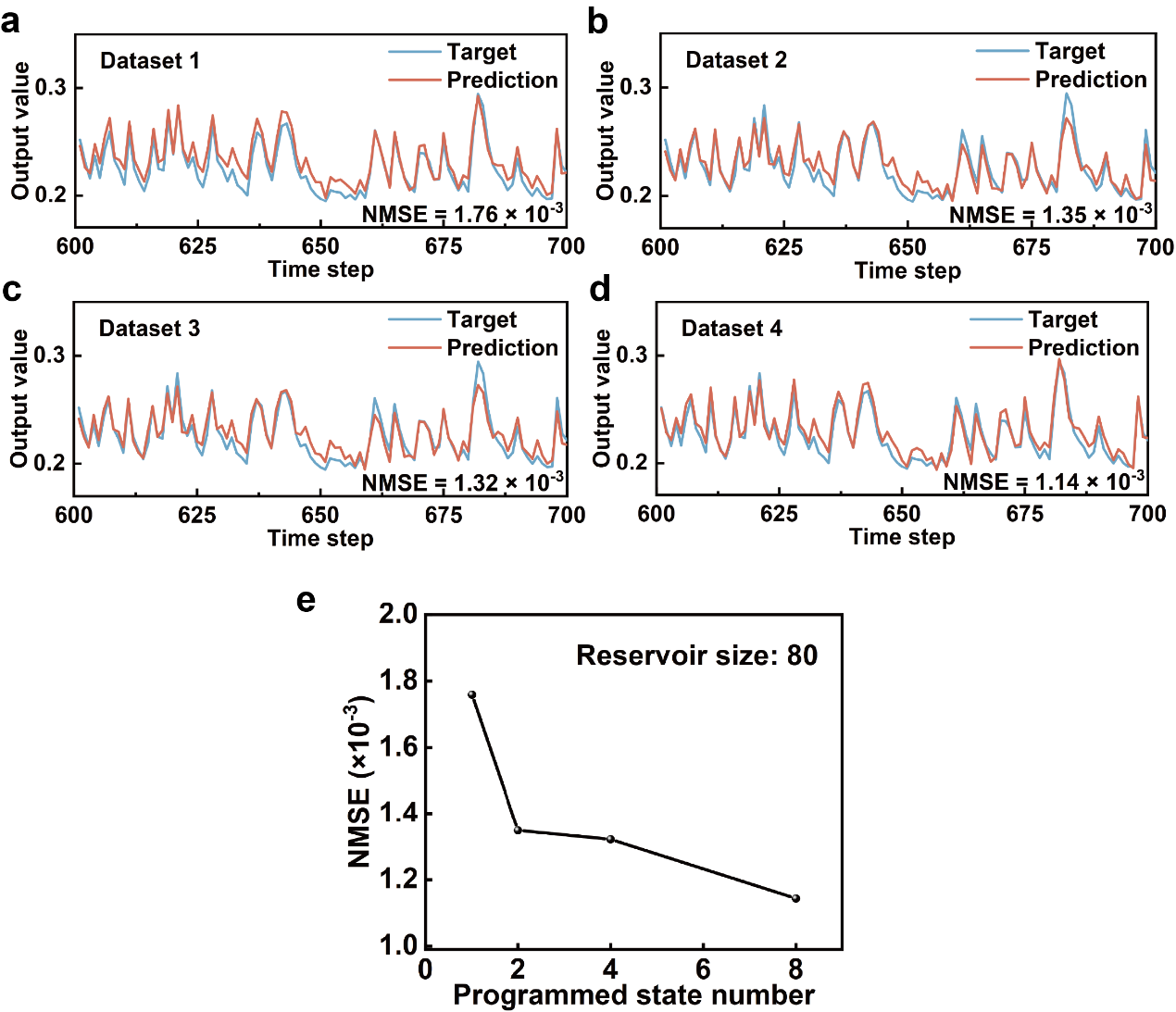
**

**Figure S17.** Prediction with a fixed reservoir size of 80. a) The prediction result of dataset 1, b) dataset 2, c) dataset 3 and d) dataset 4. e) Relationship between NMSE and the number of storage states in dataset with a fixed reservoir size of 80. With the same reservoir size, the more number of storage states, the better prediction performance of RC system. It is proved that introduction of extra storage states can improve the richness of reservoir states.

**Table S1. Test parameters of basic data (Dataset 1)**

|  | Random sequences | Programmed time (s) | Intensity range (mW cm^–2^) | Pulse width (ms) | Pulse interval (ms) |
| --- | --- | --- | --- | --- | --- |
| Basic data | $u(k)$,  $0.5-u(k)$ | 8 | 0 – 24.25 | 100 | 100 |
|  |  |  | 0 – 24.25 |  | 200 |
|  |  |  | 0 – 14.21 |  | 100 |
|  |  |  | 3.15 – 24.25 |  | 100 |

**Table S2. Test parameters of 4 kinds of dataset**

| Dataset | Possess basic data | Additional data | | | | |
| --- | --- | --- | --- | --- | --- | --- |
|  |  | Random sequences | Programmed time (s) | Intensity range (mW cm^–2^) | Pulse width (ms) | Pulse interval (ms) |
| 1 | √ | × | | | | |
| 2 |  | $u(k)$, $0.5-u(k)$ | 0 | 0 – 24.25 | 100 | 100 |
| 3 |  |  | 0, 3, 6 |  |  |  |
| 4 |  |  | 0, 2, 3, 4, 5, 6, 7 |  |  |  |

| **Table S3. A list of typical device for reservoir computing with their key features. (NMSE: normalized mean squared error; NRMSE: normalized root mean square error)** | Refs. | | this work |  | ^[7]^ | ^[8]^ |  |  | ^[9]^ | ^[10]^ | ^[11]^ | ^[12]^ |  |
| --- | --- | --- | --- | --- | --- | --- | --- | --- | --- | --- | --- | --- | --- |
|  | Accuracy | | Static: 91.13%;  Dynamic: 90.07% | NMSE 1.06 × 10⁻³ | 90% | 86.10% | 98.60% | NRMSE 0.105 | 90.77% | 80% | NRMSE 0.0096 | 88% | 98.62% |
|  | Application | | NIR handwritten digit recognition (MNIST) | Forecast: second-order nonlinear equation | DUV noisy fingerprint image recognition | Multimode handwritten digit recognition (MNIST) | Noisy QR code recognition | Forecast: MSO task | Multimode handwritten digit recognition (N-MNIST) | Handwritten digit recognition (MNIST) | Forecast: Hénon map | Multitask recognition | Dynamic hand gesture recognition |
|  | Number of reservoir states | | ≥ 11 |  | 1 | 21 |  |  | 2 | 1 | ≥ 4 | 1 |  |
|  | Regulation terminal for reservoir states | | Electrical/  Optical |  | — | Electrical/  Optical |  |  | — | — | Electrical | — |  |
|  | Optical stimulus | Response behavior | STP, PPF |  | STP, PPF | STP, PPF, PPD |  |  | STP, LTP, PPF | STP, PPF | — | STP, PPF, PPD |  |
|  |  | Optical type | 980 nm |  | 254 nm | 655 nm |  |  | 450, 532, 635 nm | 1500 nm | — | White light |  |
|  | Electrical stimulus | Response behavior | Non-volatile memory (≥ 8 levels) |  | — | STP, STD, LTP, LTD |  |  | Non-volatile memory, STP | LTP, LTD | Non-volatile memory (multilevel) | — |  |
|  | Category | | Three terminal |  | Three terminal | Three terminal |  |  | Three terminal | Three terminal | Three terminal | Three terminal |  |
|  | Device | | Au/P3HT/SiO_2_-  coated UCNPs/SiO_2_/Si |  | Au/Ti/Amorphous GaOx/SiO_2_/Si | Au/Pd/α-In_2_Se_3_/  HfO_2_/Si |  |  | Au/Cr/MoS_2_/h-BN/  Te/SiO_2_/Si | Au/Cr/Te/h-BN/Gr/  CuInP_2_S_6_/SiO_2_/Si | 55-nm NOR flash (inorganic materials) | Au/*p*-NDI/  SiO_2_/Si |  |
|  |  | | Single device | | | | | | | | | | |

| ^[13]^ |  |  | ^[14]^ |  | ^[15]^ |  |  | ^[16]^ |  | ^[17]^ | ^[18]^ |  |
| --- | --- | --- | --- | --- | --- | --- | --- | --- | --- | --- | --- | --- |
| 91.18% | Scotopic: 91.29%;  Photopic: 91.81% | 90.64% | 100% | 80.13% | 100% | 100% | > 90% | 90.45% | 97.14% | 91% | 99.97% | 100% |
| Dynamic image recognition (MNIST) | Image recognition (MNIST) | Self-adaptive mixed-illumination image recognition | 5 × 4 Digit recognition | Dynamic 4-digit classification (MNIST) | Dynamic vision recognition | Motion recognition | Intelligent traffic simulation | Handwritten digit recognition (MNIST) | Human action classification | Short sentences of language classification | Face classification | Dynamic vehicle flow recognition |
| 5 |  |  | 1 |  | 3 |  |  | 1 |  | 2 | 1 |  |
| Electrical |  |  | — |  | Electrical |  |  | — |  | — | — |  |
| STP, Threshold switching |  |  | STP |  | STP, PPF |  |  | STP, PPF, PPD |  | STP | STP, PPF |  |
| White light |  |  | 405 nm |  | Blue light |  |  | 365 nm |  | 455, 638, 725, and 811 nm | 445 nm |  |
| — |  |  | — |  | — |  |  | Reset |  | STD | — |  |
| Three terminal |  |  | Two terminal |  | Two terminal |  |  | Two terminal |  | Two terminal | Two terminal |  |
| Au/MoS_2_/Ti/  Au/Ti/SiO_2_/Si |  |  | Photodetector (MoS_2_) |  | ITO/ZnO/Nb-  doped SrTiO_3_ |  |  | Au/ZnO:N/  IGZO/TiN |  | Au/Cr/Tin sulfide/SiO_2_/Si | Au/P(VDF-TrFE)/  Cs_2_AgBiBr_6/_ITO |  |
|  | Single device | | | | | | | | | | | |

| ^[19]^ |  |  | ^[20]^ |  |
| --- | --- | --- | --- | --- |
| 88.80% | NMSE 2.08 × 10^–4^ | NRMSE 0.020 | 87.60% | NRMSE 0.128 |
| Handwritten digit recognition (MNIST) | Forecast: second-order nonlinear equation | Forecast: NARMA2 task | Handwritten digit recognition (MNIST) | Forecast: NARMA10 task |
| 8 |  |  | 24 |  |
| Mechanical (channel lengths) |  |  | Mechanical (oscillator diameter)/Electrical |  |
| — |  |  | — |  |
| — |  |  | — |  |
| STP |  |  | — |  |
| Three terminal |  |  | — |  |
| Pt/LiCoO_2_/LiSiZrO/  Pd/Pt/diamond (100) |  |  | Spin-torque nano-oscillator |  |
| Combined devices | | | | |

**Table S4. The regularization parameters λ for ridge regression training**

|  | Different reservoir size | | | | Same reservoir size | | | |
| --- | --- | --- | --- | --- | --- | --- | --- | --- |
| Dataset | 1 | 2 | 3 | 4 | 1 | 2 | 3 | 4 |
| λ | 0.11 | 0.11 | 4.5 | 0.11 | 0.11 | 2.3 | 2.4 | 0.1 |

Note: All values are the regularization parameter λ corresponding to the optimal prediction.

**Reference**

[1] Y. Zhou, S.-T. Han, Z.-X. Xu, V. A. L. Roy, *Nanotechnology* **2012**, *23*, 344014.

[2] Z. Lei, X. Ling, Q. Mei, S. Fu, J. Zhang, Y. Zhang, *Adv. Mater.* **2020**, *32*, 1906225.

[3] Y. Zhai, Y. Zhou, X. Yang, F. Wang, W. Ye, X. Zhu, D. She, W. D. Lu, S.-T. Han, *Nano Energy* **2020**, *67*, 104262.

[4] W. Namiki, D. Nishioka, T. Tsuchiya, K. Terabe, *Neuromorph. Comput. Eng.* **2024**, *4*, 024015.

[5] W. Jiang, L. Chen, K. Zhou, L. Li, Q. Fu, Y. Du, R. H. Liu, *Appl. Phys. Lett.* **2019**, *115*, 192403.

[6] C. Du, F. Cai, M. A. Zidan, W. Ma, S. H. Lee, W. D. Lu, *Nat. Commun.* **2017**, *8*, 2204.

[7] Z. Zhang, X. Zhao, X. Zhang, X. Hou, X. Ma, S. Tang, Y. Zhang, G. Xu, Q. Liu, S. Long, *Nat. Commun.* **2022**, *13*, 6590.

[8] K. Liu, T. Zhang, B. Dang, L. Bao, L. Xu, C. Cheng, Z. Yang, R. Huang, Y. Yang, *Nat. Electron.* **2022**, *5*, 761.

[9] J. Zha, Y. Xia, S. Shi, H. Huang, S. Li, C. Qian, H. Wang, P. Yang, Z. Zhang, Y. Meng, W. Wang, Z. Yang, H. Yu, J. C. Ho, Z. Wang, C. Tan, *Adv. Mater.* **2024**, *36*, 2308502.

[10] J. Zha, S. Shi, A. Chaturvedi, H. Huang, P. Yang, Y. Yao, S. Li, Y. Xia, Z. Zhang, W. Wang, H. Wang, S. Wang, Z. Yuan, Z. Yang, Q. He, H. Tai, E. H. T. Teo, H. Yu, J. C. Ho, Z. Wang, H. Zhang, C. Tan, *Adv. Mater.* **2023**, *35*, 2211598.

[11] Y. Feng, M. Tang, Z. Sun, Y. Qi, X. Zhan, J. Liu, J. Zhang, J. Wu, J. Chen, *IEEE Trans. Electron Devices* **2023**, *70*, 4972.

[12] X. Wu, S. Wang, W. Huang, Y. Dong, Z. Wang, W. Huang, *Nat. Commun.* **2023**, *14*, 468.

[13] N. Jiang, J. Tang, W. Zhang, Y. Li, N. Li, X. Li, X. Chen, R. Fang, Z. Guo, F. Wang, J. Wang, Z. Li, C. He, G. Zhang, Z. Wang, D. Shang, *Adv. Opt. Mater.* **2023**, *11*, 2300271.

[14] W. Du, C. Li, Y. Huang, J. Zou, L. Luo, C. Teng, H.-C. Kuo, J. Wu, Z. Wang, *IEEE Electron Device Lett.* **2022**, *43*, 406.

[15] H. Tan, S. Van Dijken, *Nat. Commun.* **2023**, *14*, 2169.

[16] Y. Sun, Q. Li, X. Zhu, C. Liao, Y. Wang, Z. Li, S. Liu, H. Xu, W. Wang, *Adv. Intell. Syst.* **2023**, *5*, 2200196.

[17] L. Sun, Z. Wang, J. Jiang, Y. Kim, B. Joo, S. Zheng, S. Lee, W. J. Yu, B.-S. Kong, H. Yang, *Sci. Adv.* **2021**, *7*, eabg1455.

[18] J. Lao, M. Yan, B. Tian, C. Jiang, C. Luo, Z. Xie, Q. Zhu, Z. Bao, N. Zhong, X. Tang, L. Sun, G. Wu, J. Wang, H. Peng, J. Chu, C. Duan, *Adv. Sci.* **2022**, *9*, 2106092.

[19] D. Nishioka, T. Tsuchiya, W. Namiki, M. Takayanagi, M. Imura, Y. Koide, T. Higuchi, K. Terabe, *Sci. Adv.* **2022**, *8*, eade1156.

[20] W. Jiang, L. Chen, K. Zhou, L. Li, Q. Fu, Y. Du, R. H. Liu, *Appl. Phys. Lett.* **2019**, *115*, 192403.
